# Mitochondrial modulation with leriglitazone as a potential treatment for Rett syndrome

**DOI:** 10.1186/s12967-023-04622-5

**Published:** 2023-10-26

**Authors:** Uliana Musokhranova, Cristina Grau, Cristina Vergara, Laura Rodríguez-Pascau, Clara Xiol, Alba A. Castells, Soledad Alcántara, Pilar Rodríguez-Pombo, Pilar Pizcueta, Marc Martinell, Angels García-Cazorla, Alfonso Oyarzábal

**Affiliations:** 1 Synaptic Metabolism and Personalized Therapies Lab, Department of Neurology and MetabERN, Institut de Recerca Sant Joan de Déu, 39-57 Santa Rosa Street, Esplugues de Llobregat , 08950 Barcelona, Spain; 2 Minoryx Therapeutics BE S.A., Gosselies, Charleroi, Belgium; 3 Minoryx Therapeutics S.L., Barcelona, Spain; 4 Department of Medical Genetics, Institut de Recerca Pediàtrica, Hospital Sant Joan de Déu, Barcelona, Spain; 5 Neural Development Lab, Departament de Patologia i Terapèutica Experimental, Institut de Neurociències, IDIBELL, Universitat de Barcelona, L’Hospitalet de Llobregat, Barcelona, Spain; 6 Centro de Diagnóstico de Enfermedades Moleculares, Centro de Biología Molecular Severo Ochoa, CBM-CSIC, Departamento de Biología Molecular, Institute for Molecular Biology-IUBM, Universidad Autónoma Madrid, IDIPAZ, Madrid, Spain; 7 CIBERER-Spanish Biomedical Research Centre in Rare Diseases, Madrid, Spain

**Keywords:** Rett syndrome, Metabolic modulation, Bioenergetics, Neuroinflammation, Leriglitazone, Mitochondria

## Abstract

**Background:**

Rett syndrome is a neuropediatric disease occurring due to mutations in *MECP2* and characterized by a regression in the neuronal development following a normal postnatal growth, which results in the loss of acquired capabilities such as speech or purposeful usage of hands. While altered neurotransmission and brain development are the center of its pathophysiology, alterations in mitochondrial performance have been previously outlined, shaping it as an attractive target for the disease treatment.

**Methods:**

We have thoroughly described mitochondrial performance in two Rett models, patients’ primary fibroblasts and female Mecp2^tm1.1Bird−/+^ mice brain, discriminating between different brain areas. The characterization was made according to their bioenergetics function, oxidative stress, network dynamics or ultrastructure. Building on that, we have studied the effect of leriglitazone, a PPARγ agonist, in the modulation of mitochondrial performance. For that, we treated Rett female mice with 75 mg/kg/day leriglitazone from weaning until sacrifice at 7 months, studying both the mitochondrial performance changes and their consequences on the mice phenotype. Finally, we studied its effect on neuroinflammation based on the presence of reactive glia by immunohistochemistry and through a cytokine panel.

**Results:**

We have described mitochondrial alterations in Rett fibroblasts regarding both shape and bioenergetic functions, as they displayed less interconnected and shorter mitochondria and reduced ATP production along with increased oxidative stress. The bioenergetic alterations were recalled in Rett mice models, being especially significant in cerebellum, already detectable in pre-symptomatic stages. Treatment with leriglitazone recovered the bioenergetic alterations both in Rett fibroblasts and female mice and exerted an anti-inflammatory effect in the latest, resulting in the amelioration of the mice phenotype both in general condition and exploratory activity.

**Conclusions:**

Our studies confirm the mitochondrial dysfunction in Rett syndrome, setting the differences through brain areas and disease stages. Its modulation through leriglitazone is a potential treatment for this disorder, along with other diseases with mitochondrial involvement. This work constitutes the preclinical necessary evidence to lead to a clinical trial.

**Supplementary Information:**

The online version contains supplementary material available at 10.1186/s12967-023-04622-5.

## Background

Rett syndrome (OMIM # 312750) is a neurodevelopmental disorder that affects 1 in 10,000 people, mostly girls. Usually caused by de novo mutations in the X-linked *MECP2* gene, it is a clinically and molecularly complex disorder [1]. It is characterized by a regression in neurodevelopment between 6 and 18 months of age, after normal early development. Patients experience a loss of acquired abilities, such as communication skills, and symptoms that include features of severe autism, loss of purposeful use of the hands, stereotypies, apnoea / hyperpnoea and seizures [2].

Despite several attempts, Rett syndrome still lacks a specific therapeutic option. Importantly, it was proved several years ago that the reversal of many of the features that characterize Rett syndrome is possible [3, 4], encouraging for the research of potential therapeutic options. Diverse approaches targeting specific features such as epileptic activity or breathing abnormalities have been made, and while clinical trials focused on genetic therapy are in the horizon, their efficacy and reach is dubious [5]. Considering this, the study of the Rett syndrome’s pathophysiology and the validation of new therapeutic targets and alternatives is a matter of urgency.

Among key features of the disease, such as an imbalance in excitatory/inhibitory neurotransmission and a delay in GABAergic neurotransmission maturation, mitochondrial dysfunction has been reported to play a role in Rett’s pathophysiology as well [6, 7]. Several alterations in mitochondrial homeostasis have been partially described as ultrastructure differences, decrease in electron transport chain complexes expression, with consequent reduction in ATP concentration [8]. A dysregulation in the redox state has also been described, translating into oxidative damage and lowered antioxidant defenses [9]. Metabolic and mitochondrial dysfunctions have been proposed as a potential therapeutic target. Few attempts to amend its homeostasis have been made, as through the stimulation of the brain serotonin receptor 7 (5-HT7R) with LP-211 [10] or the repurpose of known drugs such as metformin [11], used for decades in the treatment of type 2 diabetes, or triheptanoin [12], which anaplerotic function enhanced mitochondrial function in Rett models. Notwithstanding the great results in different models, none of these approaches has met successful clinical trials.

In our work we have studied mitochondrial homeostasis focusing on mitochondrial shape and bioenergetic function in both several Rett patients’ fibroblasts and female mice models. Importantly, we have determined how mitochondrial performance varies through different brain areas across neurodevelopment. Advancing from this concise description, we have evaluated the effect of a novel PPARγ-agonist, leriglitazone. It is a drug from the glitazone family that shows a superior brain penetration and a favorable safety profile, as proven in healthy controls and other neuropediatric diseases [13, 14]. Its efficacy on mitochondrial function enhancement and phenotypic amendment in Rett models endorses leriglitazone as a potential new treatment for Rett syndrome and other neurodevelopmental diseases with mitochondrial involvement.

## Methods

### Cell culturing

We used primary cultures of skin fibroblasts from patients (defined through the results section) and age-matching controls. Dermal fibroblasts were grown following standard culture conditions in minimal essential medium (MEM) supplemented with 1% glutamine, 10% fetal bovine serum (FBS) and 1% penicillin–streptomycin. Unless stated otherwise, experiments were accomplished when fibroblasts were at 80% confluence and between the 7th and 14th passages.

Leriglitazone (MW: 408.9 g/mol) was resuspended in 100% DMSO to obtain a stock solution at 10 mM concentration. We prepared a stock solution at 0.1 mM diluted in DMEM and 10% DMSO, stored at – 20 ºC for less than one month. For the working solutions, we prepare an intermediate 1 μM working solution 1% DMSO from the 0.1 mM stock solution. For leriglitazone treatment, cells were incubated for 48 h with 100 nM or 500 nM leriglitazone in the same medium, complemented with 1% DMSO.

GW9662 (M6191, Sigma-Aldrich) was used as a PPARγ antagonist. 5 mg of GW9662 were resuspended in 1.8 mL of DMSO to obtain a stock solution at 10 mM, stored at – 20 ºC. From this, we prepared an intermediate stock solution at 1 mM diluted in DMEM and 10% DMSO. Cells were treated with a working solution at 1 μM in DMEM and 1% DMSO for 72 h.

### Mice colony maintenance and sample collection

Both B6.129P2(C)-*Mecp2*^*tm1.1Bird*^/J and control littermates mice were used. To increase the translational value of the work, only female mice have been used, as they have been reported to better recapitulate the disease phenotype. Mice were housed in standard cages with ad libitum access to food and water and in controlled environmental conditions of light (12 h dark/light cycle starting at 7:30 am), temperature (22 °C) and humidity (60%). Mice were genotyped following the provider recommendations.

When stated, leriglitazone was delivered orally with the food at a dose equivalent to 75 mg/kg/day. Mice were fed pellets with or without leriglitazone from weaning until 7 months (symptomatic mice). Leriglitazone plasmatic levels were confirmed by LC–MS/MS post animal sacrifice to confirm the proper intake.

## Behavioral tests

### Novel object recognition (NOR) test

The NOR test is widely used to evaluate object recognition memory in mice. To test recognition, on the first day of the test (familiarization phase) mice were placed in a box (35 × 35 × 35 cm) with two identical objects (such as chess figures) and let freely explore the objects (meaning physically interact with them) for a total of 20 s of physical contact, or a maximum of 10 min in the cage even if the exploration time did not sum 20 s. 24 h later, on the second day of the test (test phase), mice were placed again in the box with the initial object explored during the familiarization phase and a novel object, during 10 min. The time spent exploring each object was calculated manually (exploration of an object was defined as the mouse placing its nose within 2 cm of the zone where the object located). Maximum exploration time was recorded at 20 s.

### General health score

The health scale was carried out every two weeks, from 3 months to the day of sacrifice. Mice were placed onto a laboratory bench for observation and received a score for each category (0 = absent or normal appearance; 1 = present or moderate phenotype; 2 = severe phenotype). The resulted phenotype was calculated by the sum of the scores of each mouse and then averaging all animals within each group. The categories taken into account were gait, mobility, breathing, hind limb clasping, tremors, general condition, kyphosis (i.e., increased curvature of the frontal part of the spine), fur and seizures.

## Immunofluorescence and confocal microscopy

Cells were seeded onto coverslips at 80% confluence and fixed with paraformaldehyde 4% for 20 min. Primary antibodies were used against TOMM20 (1:250, Rb; ab186735, Abcam) and α-tubulin (1:250, Ms; ab7291, Abcam) for mitochondrial network imaging, and 8-OHdG (1:250, Ms; sc-66036, Santa Cruz Biotechnology, TX, USA) for RNA oxidative damage imaging. After 1 h blocking at room temperature in a solution of 10% FBS in PBS, primary antibody was incubated for 1 h at room temperature in a solution of PBS 1% FBS. Secondary antibodies anti-Rabbit (Alexa Fluor 488, Goat; Thermo Fisher) and anti-Mouse (Alexa Fluor 546, Goat; Thermo Fisher) were incubated for 1 h at room temperature at concentrations 1:1000 in the same solution. Fluorescence was visualized using Leica TCS SP8 microscope (Leica, Wetzlar, Germany) with 63×, 1.4 N.A. immersion oil objective. Images were analyzed using Fiji software and the Mitochondria Analyzer plug-in [15].

## In vivo time-lapse imaging

Cells were incubated with 300 nM MitoTracker Green (Invitrogen) for 30 min at 37 °C and 5% CO_2_. Immunofluorescence was visualized using Leica TCS SP8 microscope (Leica, Wetzlar, Germany) with a 63x, 1.4 N.A. oil-immersion objective, 2.75 electronic zoom, and at a frame size of 512 × 512 pixels. We used a 495 nm white laser, and recorded for 2 min with 0.73 s between each frame, maintaining cell culture conditions. Mitochondrial dynamics analysis was performed with the MATLAB add-in Mitometer [16], which gives the fusion and fission number of events per track.

## Electron transmission microscopy

Mitochondrial ultrastructure was analyzed by electron microscopy. Fibroblasts were fixed in the culture plate at room temperature for 1 h with a mixture of 4% paraformaldehyde and 2% glutaraldehyde in phosphate buffer (pH 7.4) and post-fixed with 1% osmium tetroxide plus 1% potassium ferricyanide in distilled water for 1 h at 4 °C. Fibroblasts were dehydrated in ethanol and embedded in epoxy resin EML-812 (TAAB Laboratories), still attached to the culture plate. Small pieces of the thin layer of resin containing the cells were separated from the culture plate and glued to blocks of resin to cut ultrathin sections (70 nm) oriented parallel to the cell base. Sections were collected on 100-meshgrids, stained with uranyl acetate and lead citrate, and examined at 80 kV in a Jeol JEM-1010 (Tokyo, Japan) electron microscope. Images were recorded with 4 k CMOS F416 camera from TVIPS (Gauting, Germany). Electron transmission images were taken at the Electron Microscopy Service at Centro de Biología Molecular Severo Ochoa in Madrid.

Images were analysed using Fiji software measuring the following ultrastructure parameters: (1) mitochondrial major: minor axis ratio; (2) cristae length and (3) cristae width.

## Immunohistochemistry

Mice were anaesthetized with Ketamine/Xylazine at a concentration of 100 mg/kg and 10 mg/kg respectively and perfused intracardially with 4% paraformaldehyde (PFA). Brains were extracted and post-fixed in a solution of 4% PFA overnight, cryoprotected in 30% sucrose and kept frozen at − 80 °C. Sagittal sections of 40-μm thick were obtained using a cryostat, collected in a cryoprotective solution (30% glycerol, 30% ethylene glycol in 0.1 M phosphate buffer) and kept at − 20 °C until processing. For immunohistochemistry analysis, the slices were washed three times with washing solution (0.01% Triton X-100 in PBS), permeabilized with 0.25% Triton X-100 in PBS for 15 min and incubated with blocking solution (0.25% Triton X-100, 10% Horse Serum, 2% BSA) for 2 h at room temperature. Subsequently, slices were incubated with mouse anti-8-OHdG (1:1000; Santa Cruz Biotechnology), rabbit anti-calbindin (1:100; Proteintech), rabbit anti-GFAP (1:500; Dako) or rabbit anti-Iba1 (1:200; Wako) in blocking solution at 4 °C overnight. The slices were then washed with washing solution before the incubation with Alexa Fluor 488-conjugated goat anti-rabbit IgG or Alexa Fluor 546-conjugated goat anti-mouse IgG antibody (1:400; Thermo Fisher), and Hoechst (1:10,000, Thermo Fisher), in blocking solution for 2 h at room temperature. Finally, slices were washed three times with PBS and then mounted with Fluoromount Aqueous Mounting Medium (Sigma-Aldrich, MO, USA) in glass slides coated with 0.2% gelatin. Fluorescence images at 10x, 0.45 N.A. were captured using a Zeiss LSM 880 confocal microscope (Zeiss, Germany). Scales bar have been added in each representative figure.

## Western blotting

Proteins were extracted from cultured fibroblasts or mouse brain tissue through a 30’ cold incubation with RIPA and protease and phosphatase inhibitors. Following extraction, proteins were quantified by Bradford method and prepared at a homogenous concentration in Laemmli Buffer in reductive denaturing conditions and subjected to SDS-PAGE 8 or 10% and transferred to a nitrocellulose membrane at 100 V 1 h. Membranes were blocked with TBS-Tween (0.05%): milk 5% for 1 h at room temperature. Primary antibodies were incubated O/N at 4 ºC in blocking buffer, at the following concentrations: Drp1 (1:1000, Rb; ab184247, Abcam), Fis1 (1:500, Ms; sc-376469, Santa Cruz Biotechnology), OPA1 (1:2000, Ms; BD-612607, BD Biosciences), Mfn2 (1:500, ab124773, Rb; Abcam), MnSOD (1:80,000, Rb; ADI-SOD-111-F, Enzo Life Sciences) and GPx1 (1:1000, Rb; ab108427, Abcam). Vinculin (1:20,000, Ms; sc-59803, Santa Cruz Biotechnology) was used as a loading control. For the OXPHOS complexes detection, proteins were denatured in a non-reductive buffer, and incubated after SDS-PAGE and Western blot with OXPHOS Human WB Antibody Cocktail (1:1000, Ms; 45-8199, Thermo Fisher). The secondary antibodies used were HRP-conjugated goat anti-Rabbit and goat anti-Mouse IgG antibodies (Thermo Fisher) and were detected using the Pierce^TM^ ECL Western Blotting substrate (Thermo Fisher). Quantification of protein expression was performed using Fiji software by calculating protein densitometry relative to controls and normalized to vinculin.

## RT-qPCR

RNA for RT-qPCR was extracted using RNeasy Fibrous Tissue Mini Kit (Qiagen), following the manufacturer’s instructions. The total RNA was eluted in 40 µL of RNAse-free water and stored at − 80 °C. The RNA concentration was measured using the NanoDrop 2000 Spectrophotometer (Thermo Fisher). qPCRs were carried out following a two-step protocol. First, cDNA was synthesized from a total of 500 ng of RNA per reaction, following the recommendations provided with SuperScript III First-Strand Synthesis SuperMix for RT-qPCR (Invitrogen). After the RT-PCR reaction, the cDNA from control and Rett fibroblasts was pooled. Second, qPCR was performed in a QuantStudio 6 Flex Real Time PCR System (Thermo Fisher) with PowerUp SYBR Green Master Mix (Thermo Fisher). The data were analyzed using a comparative method, correlating the initial template concentration with the cycle threshold (Ct) to obtain the relative quantity (RQ) of the RNA. The RQ is defined as 2^−∆∆Ct^, where ∆∆Ct is the ∆Ct of the patient cell line minus the ∆Ct of the control cell line, and ∆Ct is the Ct of the target gene minus Ct of the endogenous gene (*RPLP0*).

Primers used for determination of mitochondrial mass were: *mtND1, CO1, CO2, CO3* and *HK2*. Primers used for detection of PPARγ pathway and known to be altered in Rett were: *PGC1a, PKM, MnSOD, SREBF2.*

## ATP content measurement

ATP concentration was evaluated both in fibroblasts and mice brains.

In the former, cells were harvested and resuspended in boiling ATP extraction buffer (100 mM Tris–HCl, 4 mM EDTA pH 7.7). Finally, ATP concentration was assayed by bioluminescence using a luciferin-luciferase system (ATP Bioluminescence Assay Kit CLS II, Roche) according to manufacturer's instructions. ATP concentration was corrected per mg of protein. To discriminate between glycolytic and non-glycolytic ATP, cells were grown either in complete MEM 10% FBS or supplemented with the hexokinase inhibitor 2′-deoxy-d-glucose (D8375, Sigma-Aldrich) at a concentration of 100 mM for 2 h prior to extraction.

For analysis of ATP concentration in different brain areas, brains were dissected, and samples were kept in cryopreservation buffer at − 80 °C until experiment. Samples were weighted, homogenized in pre-cooled 10% HClO_4_, neutralized with KOH 2.5 M and the supernatant diluted in 100 mM Tris–4 mM EDTA [17]. Total ATP was detected by luciferin–luciferase bioluminescence assay (ATP Bioluminescence Assay Kit CLS II, Roche). ATP concentration was corrected by mg of tissue.

## Oxygen consumption rate

Oxygen consumption rate (OCR) was measured using the XF24 Extracellular Flux Analyzer (Seahorse Bioscience, Izasa Scientific). 80,000 fibroblasts per well were seeded in XF 24-well cell culture microplates (Seahorse Bioscience) in 250 μL of MEM 10% FBS and incubated in normal conditions for 24 h in MEM containing 1 g/L of glucose. Two hours previous to the assay, the growth medium was replaced with 700 μL of fresh medium, allowing the media temperature and pH to reach equilibrium before the first measurement. After an OCR baseline measurement, 50 μL of oligomycin, carbonyl cyanide-4-(trifluoromethoxy) phenylhydrazone (FCCP), rotenone and antimycin solutions were sequentially added to each well to reach working concentrations of 6 μM, 50 μM, 1 μM and 1 μM respectively. Results were normalized per cell counting after the experiment and normalized to 30,000 cells. To determine Oligomycin Sensitive Respiration (OSR) we calculated the difference between oxygen consumption before and after adding oligomycin to the medium, considered as ATP-linked respiration.

## Analysis of intracellular O2^·−^ by flow cytometry

Superoxide anion concentration was measured using MitoSOX™ Red probe (M36008, Invitrogen). Alive fibroblasts were incubated with the dye at a concentration of 5 μM for 10 min at 37 °C according to manufacturer's instructions. Cells were subsequently resuspended in complete Hanks Balanced-Buffered Saline (HBSS) and analyzed by flow cytometry in a NovoCyte Flow Cytometer (ACEA Biosciences).

## TBARS assay

Lipid peroxidation in brain samples was detected using a colorimetric assay (ab118970, Abcam) in which the malonaldehyde (MDA) present in the samples reacted with thiobarbituric acid (TBA) to form the MDA-TBA adduct. In brief, samples were homogenized in 303 μL lysis solution (MDA lysis buffer + BHT) for hippocampus and cerebellum, and 606 μL for cortex, and centrifuged at 13,000*g* for 10 min. 200 μL of supernatant were collected and added 600 μL of TBA reagent. Samples were incubated at 95 ºC for 30 min and cooled to room temperature previous to absorbance measurement at 532 nm. Measurements were corrected by mg of protein.

## Cytokines multiplex

A panel of thirteen cytokines (IL-1α, IL-1β, IL-6, IL-10, IL-12p70, IL-23, IL-27, IFN-β, IL-17A, MCP-1, TNF-α, IFN-γ, GM-CSF) was detected by flow cytometry using an immunoassay based on bead-fluorescence detection on a V-bottom plate (#740446, BioLegend) according to the manufacturer’s guidelines. For sample preparation, samples were weighted, homogenized with lysis buffer (PBS 1×, NP-40 1% and 1× protease inhibitor) as specified in [18], incubated with rotation for 1 h at 4 ºC, sonicated and centrifuged at 14,000 rpm (19,500*g*) for 15 min at 4 ºC. Supernatant was collected and centrifuged at 14,000 rpm (19,500*g*) for 15 min at 4 ºC. In brief, 25 μL of sample were added to 25 μL of assay buffer and 25 μL mixed beads and incubated for 2 h shaking at room temperature. Beads were spun down, washed and 25 μL of detection antibodies were added and incubated for 1 h shaking at room temperature. Then 25 μL of SA-PE were added and incubated for 30 min shaking at room temperature. Finally, beads were spun down, samples were added 150 μL of wash buffer and were read on a flow cytometer. Measurements were corrected by mg of protein.

## Statistical analysis

GraphPad Prism 9.0 (La Jolla, CA, USA) software was used for statistical analysis. First, outliers are identified by ROUT test and discarded, and then data normality was determined. ANOVA for parametric data or Kruskal–Wallis for non-parametric data were performed, with posterior multiple comparisons analysis correction when appropriate. Equal standard deviations were not assumed. Two-way ANOVA was used for comparing groups with two or more variables. Results are represented as mean + individual values, mean ± SD, lower to upper quartile box + individual values, or min to max box plot with line at mean. RStudio was used for histogram representation.

Differences were considered as statistically significant at *p* < 0.05, and coded through the figures as follows: *p < 0.05, **p < 0.01, ***p < 0.001, and ****p < 0.0001.

## Results

### Mitochondrial shape and bioenergetic functions are altered in Rett syndrome fibroblasts

Within the multiple physiological routes affected in Rett syndrome, mitochondrial dysfunctions had been previously suggested. We have studied both mitochondrial shape (ultrastructure and network dynamics) and bioenergetic homeostasis (energy metabolism and oxidative stress) in fibroblasts from eight Rett patients with different *MECP2* mutations and different severity presentations of the disease (Additional file 1: Table S1), compared to four different healthy controls.

**Fig. 1:**
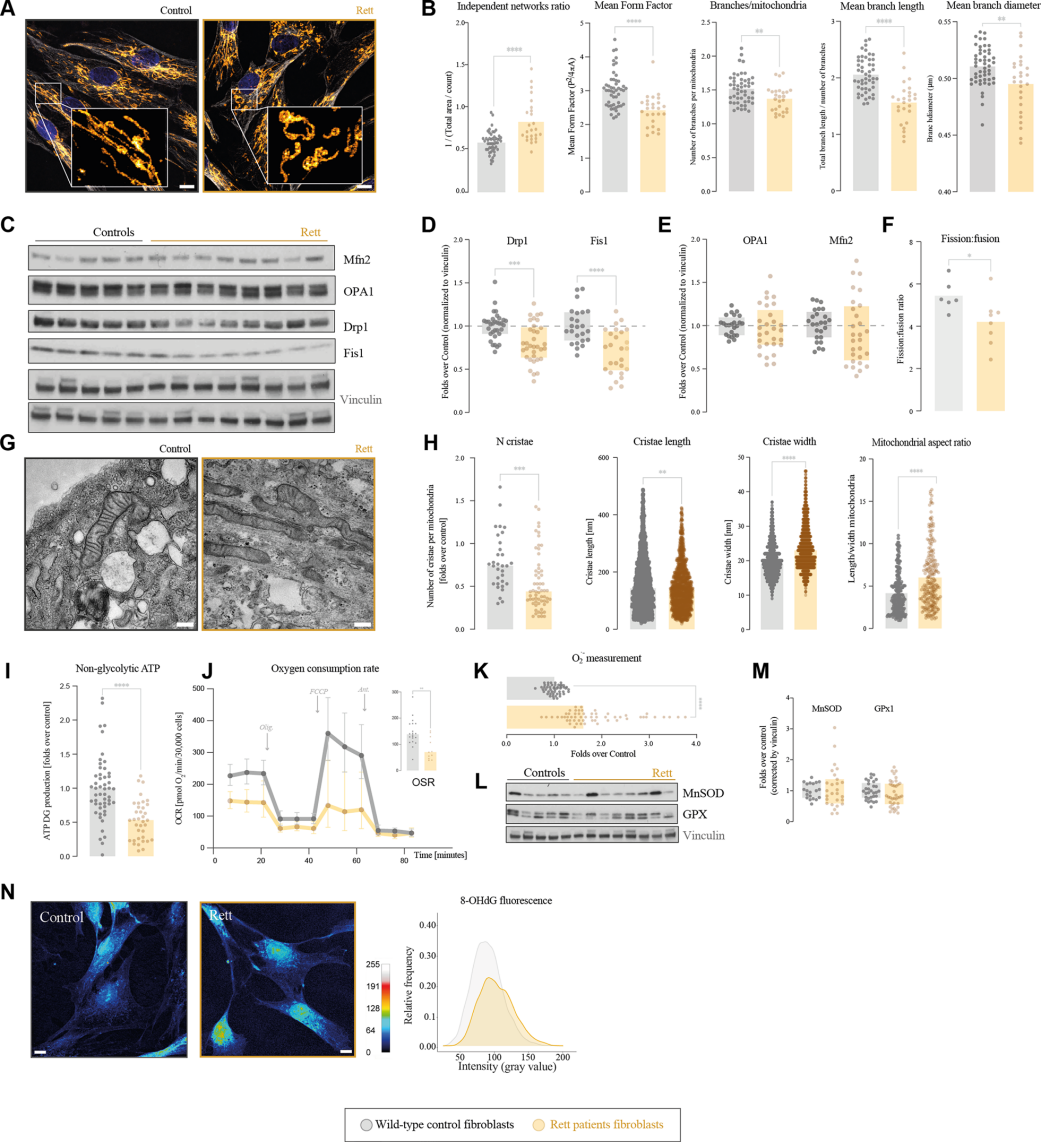
Rett syndrome primary fibroblasts recapitulate alterations in mitochondrial homeostasis. We have focused the analysis on mitochondrial shape (**A**–**H**) and energy production (**I**–**N**). Mitochondrial network shape was studied by immunofluorescence of mitochondrial marker TOMM20 and parametrization with “Mitochondria Analyzer” plugin; **A** representative image of mitochondrial network IF (TOMM20 in yellow, nuclei in blue, and tubulin in grey; scale bar 10 µm; 6× zoomed section included in the white box) and **(B)** parametrization of number of independent networks corrected by area and mean form factor (a shape measure given by: P^2/(4piA): 1 indicates round object and increases with elongation, expressed as mean FF of objects in image), number of branches per mitochondrial network, and mean branch length and diameter. **C**–**E** Analysis and densitometry quantification of mitochondrial dynamics associated proteins Mfn2, OPA1, Drp1 and Fis1 by Western blot and corrected by vinculin. **F** Quantification of the fission and fusion events from in vivo imaging of mitochondrial network. Analysis of mitochondrial ultrastructure was performed by transmission electron microscopy; **G** representative images (scale bar 300 nm) and **H** parametrization of number of cristae per mitochondria, cristae length and width, and mitochondrial major:minor axis ratio. **I** Non-glycolytic ATP production –assumed as mitochondrial ATP– measured by luciferin-luciferase luminescence upon incubation with 2-deoxyglucose. **J** Mitochondrial respiration profile was measured by Seahorse in the presence of oligomycin, FCCP and Antimycin A. Oligomycin Sensitive Respiration (OSR) was calculated as the difference of oxygen consumption before and after adding oligomycin (considered as ATP-linked respiration). **K** O_2_^.−^ production measured by flow cytometry with the MitoSOX probe **L** Expression of antioxidant enzymes MnSOD and GPX by Western blot, and **M** quantification of densitometry corrected by vinculin. **N** Oxidative damage was detected based on the fluorescence of RNA oxidative damage marker 8-OHdG, showed with the intensity-revealing LUT “royal” (calibration bar shown at the lower right corner of the panel), at 63× magnification; scale bar represents 10 µm, and represented as relative frequency of intensity values, calculated with Fiji software. All experiments were done in at least two different Rett and control cell lines, with three technical replicates and each experiment was done in triplicates. Statistical analyses were performed as described in Materials and Methods. *p < 0.05, **p < 0.01, ***p < 0.001, ****p < 0.0001. Absence of asterisk means no statistically significant difference. Control fibroblasts in grey; Rett patients’ fibroblasts in yellow

We first analyzed mitochondrial network by immunofluorescence of the mitochondrial marker TOMM20, and parametrization of its structure with Mitochondria Analyzer algorithms. It revealed alterations in mitochondrial grid number and morphology, as Rett mitochondrial networks were less interconnected (higher number of independent networks) and less ramified (Fig. 1A, B) than controls. While they summarize the general picture, these results were, however, not homogeneous within all patients, and some individualities have been found along the different cell lines, as shown in Additional file 1: Fig. S1A.

As mitochondrial network maintenance is closely regulated through mitochondrial dynamics, we upraised the expression of fission and fusion-mediating proteins through Western blot. We registered a significant decrease in the expression of both proteins involved in mitochondrial fission, Drp1 and Fis1 (Fig. 1C, D). On the contrary, we did not detect changes in the proteins responsible for the fusion processes OPA1 or Mfn2, (Fig. 1C, E), suggesting a reduction in the fission: fusion rate. This was corroborated by in vivo time-lapse image acquisition of mitochondria, stained with the probe MitoTracker^TM^ and visualized by confocal microscopy, recording during 2 min with 0.7 s between each frame. Subsequent analysis of the fusion and fission events with the MATLAB add-in Mitometer confirmed a decrease in the fission rate of Rett mitochondria (Fig. 1F and Additional file 2, 3: Videos S1, S2). These results are compatible within each other and support that Rett fibroblasts bear elongated and non-fragmented mitochondria.

Next, we analyzed mitochondrial structure differences between Rett fibroblasts and controls through imaging by Transmission Electron Microscopy (TEM). Agreeing with the previous results [19], we found a hyper fused mitochondria phenotype, as the major: minor axis ratio was higher in Rett mitochondria. On top of that, Rett fibroblasts’ mitochondria showed some structural abnormalities regarding cristae, such as a reduction in their number, which were also shorter and wider than control ones (Fig. 1G, H).

Since mitochondrial form and function follow each other, we next analyzed the mitochondrial bioenergetic performance, focusing on ATP production, respiratory capacity, and Reactive Oxygen Species (ROS) generation and metabolism. To discriminate between total ATP and non-glycolytic (and hence mitochondrial) ATP, we measured ATP concentration in the presence of the glycolysis inhibitor 2-deoxy-d-glucose, revealing that Rett mitochondria produced half the ATP controls did (Fig. 1I). For its corroboration, we measured real-time oxygen consumption rate (OCR) of Rett and control fibroblasts, revealing an overall reduction in the OCR in the MeCP2 deficient cells. Rett fibroblasts showed a lowered respiratory capacity in both basal and maximal respiration, calculated after the addition of the uncoupler FCCP. Moreover, they also showed a significant reduction in the Oligomycin-Sensitive Respiration (OSR), this is, the fraction of oxygen consumed for ATP production (Fig. 1J). These results were consistent with an impairment in mitochondrial bioenergetic function.

Mitochondrial respiration defects often result in ROS generation, that can end in oxidative damage. Therefore, we quantified the ROS superoxide anion (O_2_^·−^) by detection through flow cytometry and using the probe MitoSOX. We recorded that Rett fibroblasts were exposed to two-fold higher concentrations of superoxide anion compared to controls (Fig. 1K). Surprisingly, we did not detect any differences in the expression of the antioxidant enzymes MnSOD or GPX between Rett and control fibroblasts (Fig. 1L, M). Moreover, this increase in oxidative stress resulted in an enhanced oxidative damage in Rett cells, measured by the detection of 8-OHdG, (Fig. 1N), a widely accepted marker of oxidative damage easily quantifiable by immunofluorescence.

We have worked with 8 Rett lines and observed different degrees of harshness among patients specially regarding mitochondrial network shape and antioxidant defense, both the decrease in mitochondrial ATP production and the increase in ROS and oxidative damage were consistent along the studied cell lines (Additional file 1: Fig. S1B, C). These differences were not mutation-dependent, as patients bearing the same mutations showed differences in mitochondrial performance. Skin fibroblasts are a useful and cost-effective resource to investigate mitochondrial abnormalities in neurodevelopmental disorders [20, 21].

Altogether, our data supported an alteration in mitochondrial form and function.

### Bioenergetic dysfunction is also detectable in Rett syndrome female mice models, displaying differences among brain areas and already evident at pre-symptomatic stages

Encouraged by the alterations found in fibroblasts, we moved towards the analysis of mitochondrial dysfunction in the Rett animal model, wondering whether the dysfunction was homogeneous through the different brain areas and steady through neurodevelopment. For that, we studied female mice from the B6.129P2(C)-*Mecp2*^*tm1.1Bird*^/J strain, as they recapitulate many elements of the disease. Building on previous reports on mitochondrial dysfunction in whole brain samples, we have analyzed three different brain areas separately- cortex, hippocampus and cerebellum, regarding ATP concentration and oxidative stress (assessed through lipid peroxidation). Interestingly, as patients do, female mice also go through an early normal or non-phenotypic phase, and only after a few months develop the full Rett phenotype. Therefore, we have brought neurodevelopment into the equation, studying differences at two developmental stages: pre-symptomatic (3 months old) and fully symptomatic (7 months old) mice.

**Fig. 2:**
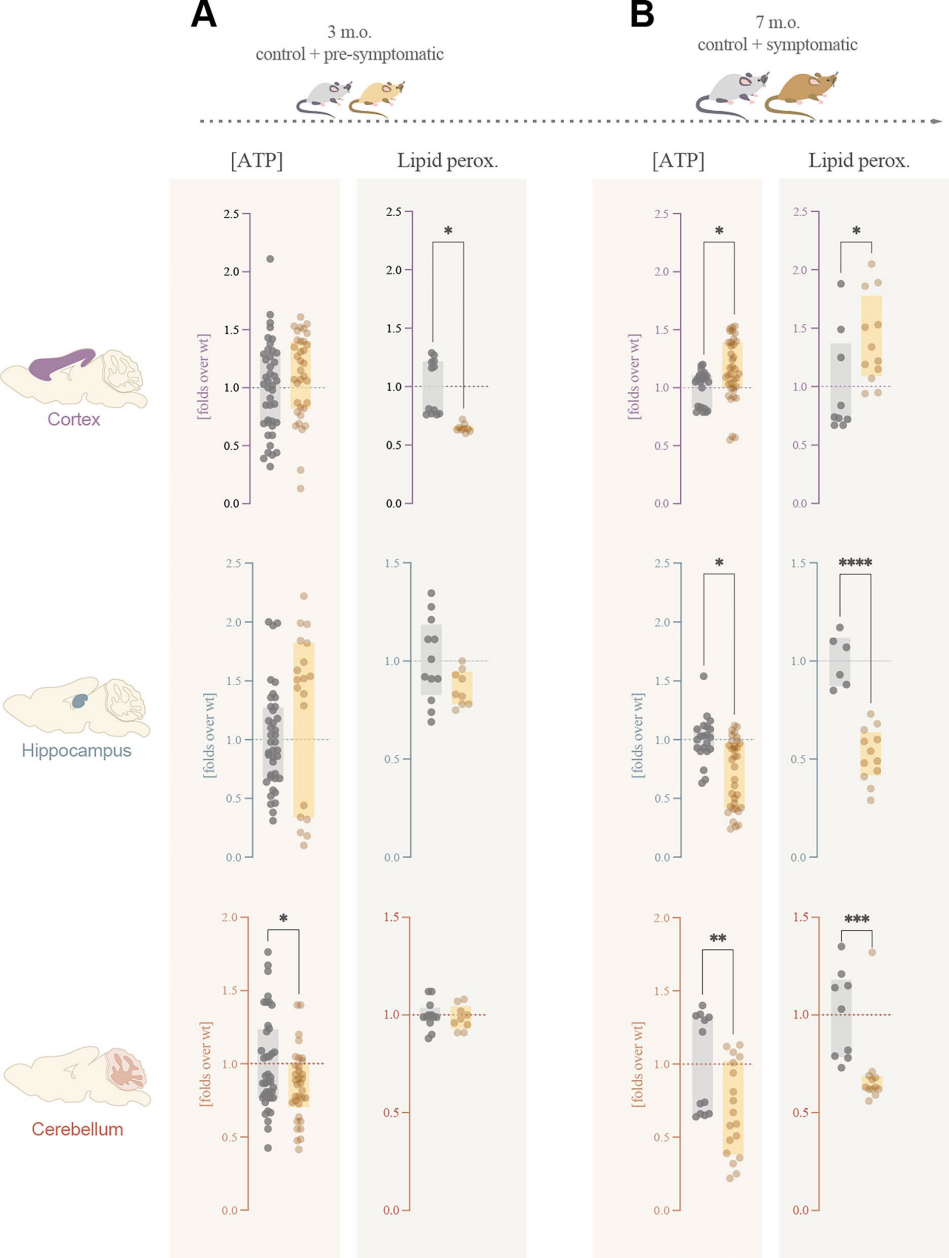
Bioenergetic characterization of pre-symptomatic (3 m.o.) and symptomatic (7 m.o.) Rett mice. Given the time-dependent character of Rett syndrome, we studied both wild-type and Rett mice at two development time points: **A** pre-symptomatic (3 months) and **B** post-symptomatic (7 months) brains. At the time points, mice brains were dissected and subjected to analysis. ATP concentration by luciferin-luciferase luminescence and lipid peroxidation by TBARS were analysed in three brain areas: cortex, hippocampus and cerebellum. All experiments were done in at least three different mice, with three technical replicates. The ATP production experiment was done in triplicates, while lipid peroxidation was a unique experiment. Statistical analyses were performed as described in Materials and Methods. *p < 0.05, **p < 0.01, ***p < 0.001, ****p < 0.0001. Absence of asterisk means no statistically significant difference. Wild-type female mice in grey; Rett female mice in yellow

Pre-symptomatic mice showed already alterations in both ATP concentration and lipid peroxidation, which were brain area-specific (Fig. 2A): cerebellums from Rett mice had a decreased concentration of ATP, which was within control values in hippocampus and cortex samples. Lipid peroxidation displayed differences as well, as we observed a significant decrease in pre-symptomatic Rett cortex samples along with non-significant differences in hippocampus and cerebellum.

As expected, the variations in these two parameters were more evident when we moved into symptomatic 7 months old mice brains (Fig. 2B). The observed reduction in cerebellum ATP concentration of pre-symptomatic mice was continued in the symptomatic ones, and also evident in Rett hippocampus at this stage. Opposite to that, an increase in [ATP] was detected in Rett cortex, suggesting that alterations are dynamic both between brain areas and through neurodevelopment. ATP concentration is also dynamic through time, and while Rett and control samples varied analogously throughout development, the differences between Rett and WT increased over time (Additional file 1: Fig. S2). On the other hand, a decrease in lipid peroxidation was detected in both hippocampus and cerebellum of symptomatic mice, opposite to a significant increase observed in Rett mice cortex.

### Leriglitazone corrects bioenergetic but not dynamics alterations in Rett fibroblasts

**Fig. 3:**
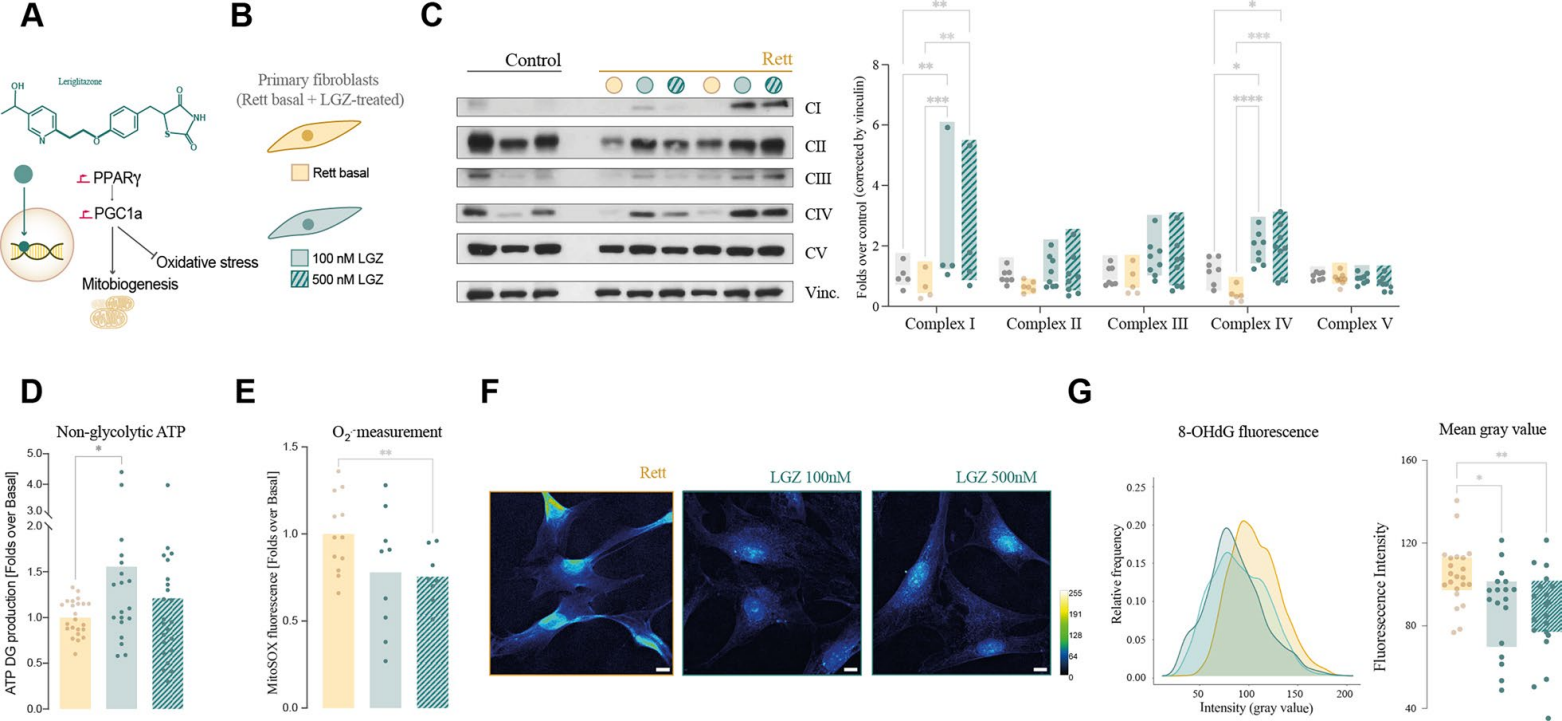
Correction by leriglitazone of both bioenergetics and oxidative stress in Rett fibroblasts. **A** Schematic representation of leriglizatone (LGZ) and its activity as a PPARγ agonist in a cell. LGZ binds to PPARγ, activating the expression of the genes under its regulation through interaction with PGC1α. **B** LGZ effect was evaluated first on Rett fibroblasts at two concentrations, 100 nM and 500 nM for 48 h. **C** PPARγ pathway activation has an effect on the detection of all electron-transport chain complexes, detected by Western blot and densitometry quantification corrected by vinculin. The ultimate goal of LGZ treatment was to correct the previously described alterations, observing significant corrections in ATP (**D**), ROS production (**E**) and (**F**) oxidative damage measured in terms of 8-OHdG detection. Images are shown with the intensity-revealing LUT “royal”, at a 63× magnification; scale bar represents 10 µm. Images were quantified and statistical significance of corrections was evaluated (**G**). All experiments were done in at least two different control and Rett cell lines, with three technical replicates and each experiment was done in triplicates. Statistical analysis was performed as described in Materials and Methods. *p < 0.05, **p < 0.01, ***p < 0.001, ****p < 0.0001. Absence of asterisk means no statistically significant difference. Control fibroblasts in grey; Rett patients’ fibroblasts in yellow; LGZ-treated Rett patients’ fibroblasts in green

Given the mitochondrial alterations in the studied models, we next investigated if these dysfunctions could be corrected with leriglitazone (LGZ). Leriglitazone is a selective peroxisome proliferator-activated receptor gamma (PPARγ) agonist that can efficiently cross the blood–brain barrier (BBB) [13]. Activation of the PPARγ/PGC1α pathway stimulates mitochondrial biogenesis, reduces oxidative stress and improves mitochondrial function (Fig. 3A). Leriglitazone has demonstrated preclinical efficacy in the correction of the phenotype in in vitro and in vivo models of other neurodevelopmental diseases such as X-linked adrenoleukodystrophy (X-ALD)[13] and Friedreich’s ataxia (FA) [22] and clinical benefit for X-ALD and FA patients in clinical trials [14, 23].

We initially investigated the effect of leriglitazone in Rett fibroblasts incubating the cells at two different concentrations, 100 and 500 nM, for 48 h (Fig. 3B). The target engagement of LGZ treatment was assessed by measuring the expression of four genes under PPARγ regulation (*PGC1α, PKM, MnSOD* and *SREBF2*), that represent four key pathways under its modulation. Analysis by qPCR revealed an increase in the transcripts’ detection upon LGZ incubation, verifying that leriglitazone effectively modulated PPARγ-signaling pathway in the patients’ fibroblasts (Additional file 1: Fig. S3A). Leriglitazone treatment resulted also in an increase in the expression ratio of *mtND1*, *CO1*, *CO2* and *CO3* corrected over *HK2*, pointing towards an increase in the expression of respiratory complexes (Additional file 1: Fig. S3B). These results were confirmed by anti-OXPHOS complexes Western blot, as all four complexes expression was increased upon treatment with Leriglitazone (Fig. 3C), being statistically significant the increases in complex I and IV. This was paired to an increase in the expression of several mitochondrial mass markers (Additional file 1: Fig. S2C).

Upon confirmation of the effective PPARγ-signaling pathway, we evaluated leriglitazone’s efficacy in the correction of the described mitochondrial alterations in Rett fibroblasts. Treatment with leriglitazone resulted in a 50% increase in the production of mitochondrial ATP, suggesting an enhancement on mitochondrial energy production in Rett fibroblasts (Fig. 3D). Importantly, these changes were accompanied by a significant 20% decrease in superoxide anion concentration in LGZ-treated cells (Fig. 3E), and a reduction of oxidative damage in Rett fibroblasts, evidenced by a lower detection of 8-OHdG fluorescence intensity (Fig. 3F, G). These changes were not associated to a significant change in antioxidant enzymes expression (Additional file 1: Fig. S3D).

Opposite to the positive effects regarding the bioenergetic performance, we did not observe a recovery in mitochondrial network shape or dynamics, as neither network structure (measured by anti-TOMM20 immunofluorescence), expression of dynamic markers (measured by Western blot) or fusion and fission events (analyzed by in vivo imaging) showed significant differences after treatment with leriglitazone (Additional file 1: Fig. S4; Additional files 3, 4: videos S2, S3).

To confirm that these changes were a direct consequence of PPARγ pathway activation, we treated the cells with the PPARγ-signaling inhibitor GW9662 at a concentration 1 μM for 72 h. Pathway inhibition blocked the effect of leriglitazone regarding all the observed measures. As shown in Additional file 1: Fig. S5A, ATP production was even decreased when Rett fibroblasts were treated with either GW9662 or a combination of leriglitazone + GW9662. Analogously, superoxide anion production was not reduced after treatment with leriglitazone + GW9662 Additional file 1: Fig. S5B). These results confirmed the beneficial effect of PPARγ pathway activation in the recovery of the bioenergetic deficits in Rett syndrome fibroblasts.

### Leriglitazone corrects the bioenergetic alterations in symptomatic Rett female mice, together with the amelioration of their phenotypic outcome

**Fig. 4:**
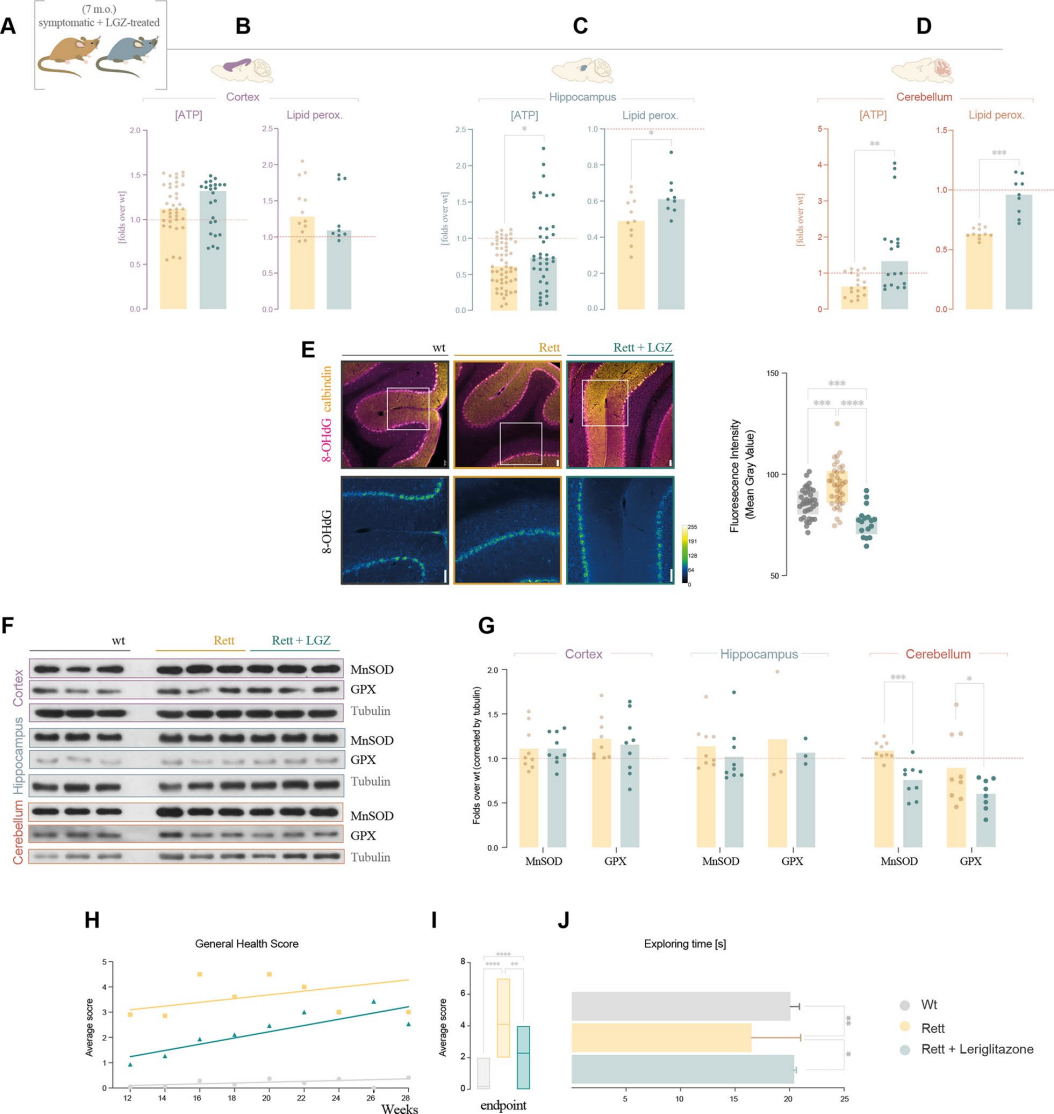
Correction by LGZ of the bioenergetic component in hippocampus and cerebellum, together with a phenotypic amelioration in symptomatic Rett mice. **A** Treatment with LGZ was assayed in Rett mice, treating them from weaning until sacrifice at the symptomatic stage (7 m.o.). Both ATP concentration (detected by bioluminescence) and lipid peroxidation (detected by TBARS) were assayed in untreated and LGZ-treated Rett mice in **B** cortex, **C** hippocampus and **D** cerebellum. **E** to **G** show results regarding oxidative stress and antioxidant markers in the brain areas of interest. **E** Detection of the RNA oxidative damage marker 8-OHdG in cerebellum by immunofluorescence. 8-OHdG staining is shown in magenta and calbindin in yellow for Purkinje cells in the upper images at 10× magnification; in the lower images 8-OHdG is shown with the intensity-revealing LUT “Green Fire Blue”, with a 2× electronic zoom; scale bar represents 50 µm in both images. **F** Antioxidant markers MnSOD and GPX in cortex, hippocampus and cerebellum detected by western blot, and their respective densitometry quantification, normalized by tubulin (**G**). Phenotypical characterization of the treated mice, registering an improvement in the General Health Score (**H**), which was maintained until the endpoint (**I**) and a recovery of the explorative behaviour of Rett mice upon treatment in the NOR-test (**J**). All experiments were done in at least three different mice, with three technical replicates and each experiment was done in triplicated (except for lipid peroxidation). Statistical analysis was performed as described in Materials and Methods. *p < 0.05, **p < 0.01, ***p < 0.001, ****p < 0.0001. Absence of asterisk means no statistically significant difference. Wild-type female mice in grey; Rett female mice in yellow; LGZ-treated Rett female mice in green

Encouraged by the positive results in fibroblasts and the description in animal models, we then studied the effect of leriglitazone in the Rett animal model (Fig. 4A). The drug was orally administered mixed with the diet at a dose equivalent to 75 mg/kg/day from weaning and until analysis at 7 months, and its intake was controlled at sacrifice through quantification of leriglitazone concentration in plasma by LC–MS/MS (Additional file 1: Fig. S6).

Both ATP concentration deficit and lipid peroxidation profile were corrected in hippocampus and cerebellum, with no significant changes observed in cortex (Fig. 4B–D). These corrections were especially noticeably in cerebellum, as the increase in ATP concentration reached levels over the control values, and lipid peroxidation was normalized with respect to control mice.

The results regarding lipid peroxidation were opposite to the expected, as we initially predicted an increase in lipid peroxidation and subsequent correction through treatment with leriglitazone. However, lipid metabolism has been described to be altered in several Rett syndrome samples [24, 25], thus alterations in lipids profiles could be contributing to the counterintuitive interpretation of the results. On top of that, PPARγ activation regulates lipids biosynthesis and we have demonstrated that leriglitazone is effectively regulating the expression of the cholesterol master-regulator *SREBF2.* To by-pass the effect of lipid metabolism in the assessment of oxidative stress, we measured oxidative damage through the quantification of 8-OHdG. Rett mice cerebellum showed increased levels of 8-OHdG, that were within control values in leriglitazone-treated mice (Fig. 4E; Additional file 1: Fig. S7).

To deepen the analysis of the effect of leriglitazone in the management of oxidative stress, we analyzed the expression of the ROS-metabolizing enzymes MnSOD and GPX in LGZ-treated Rett mice by Western blot (Fig. 4F). As happened in the ATP concentration and lipid peroxidation corrections, the most significant effect of leriglitazone was a 20 to 30% decrease in both MnSOD and GPX expression in cerebellum (Fig. 4G). These results outstand cerebellum not only as one of the most affected areas regarding bioenergetic functions but also as one of the most leriglitazone-respondent areas in Rett animal models.

Finally, to assess the ultimate effect of leriglitazone as a potential drug for the treatment of Rett syndrome, we studied its effect on Rett mice phenotype correction. Leriglitazone effectively ameliorated the overall mice status, assessed through the General Health Score, a scale that evaluates the most relevant features as mobility, breathing abnormalities, tremor or physical deterioration. The treated mice developed a milder form of the disease, which difference was evident from the beginning of the assessment (12 weeks) (Fig. 4H), and especially significant in fully symptomatic mice (7 months old) (Fig. 4I). On top of that, treated mice increased the exploring time, measured as time spent analyzing objects in Novel Object Recognition Test (NORT), suggesting an enhanced explorative behavior (Fig. 4J).

### Neuroinflammatory component underlies in Rett syndrome, which is prevented with leriglitazone

**Fig. 5:**
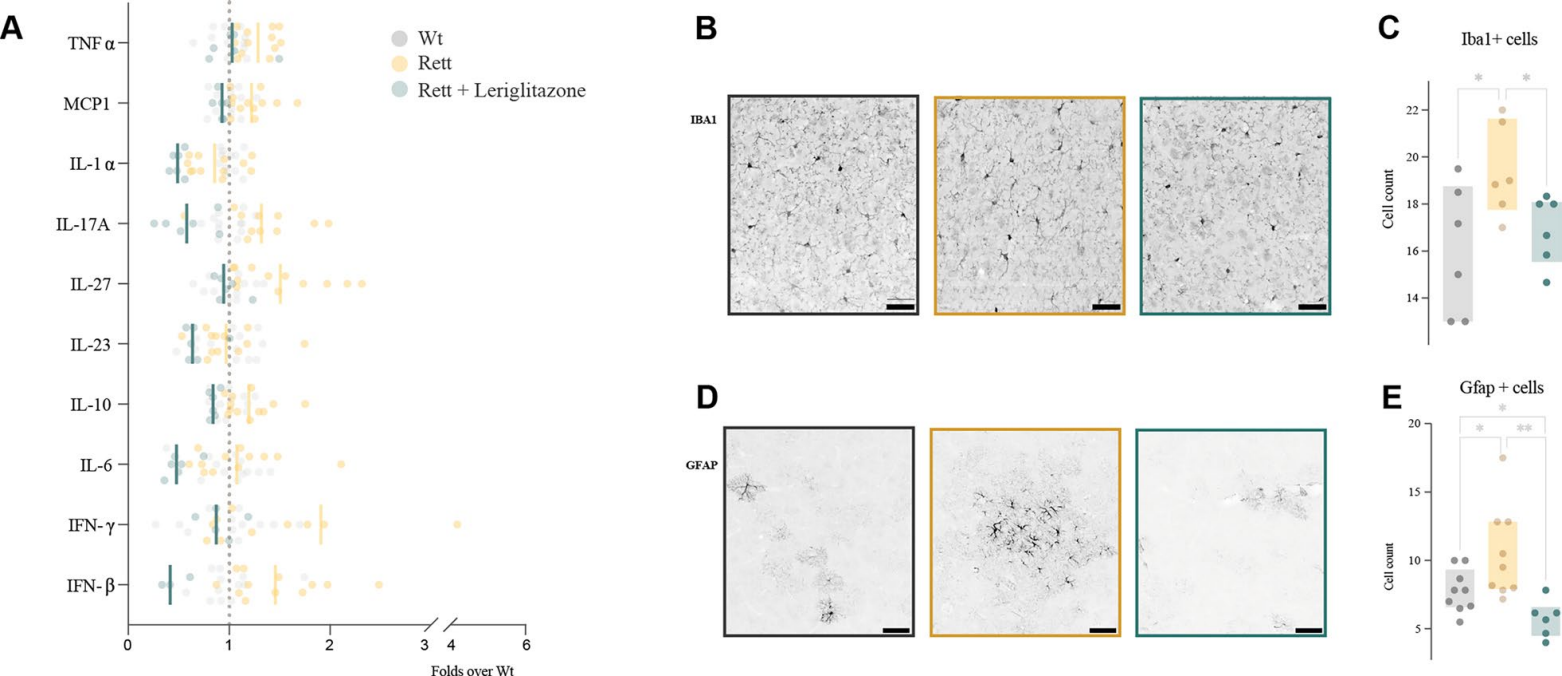
Neuroinflammatory component in cerebral cortex of symptomatic Rett mice is corrected after leriglitazone treatment. **A** Rett cerebral cortex at 7 m.o. showed a pattern of increased cytokines expression detected by flow cytometry, that was corrected in leriglitazone-treated mice (Nested one-way ANOVA analysis; *p*-value Rett *vs* wt = ** 0.0016; Rett *vs* Rett + leriglitazone = **** < 0.0001) **B** Iba1 and **D** GFAP cell-positive number were appraised, as microglia and astrocytic specific markers, respectively. **C**, **E** Quantifications of Iba1- and GFAP-positive cells revealed an increase in microglia and active astrocytes in symptomatic Rett mice cortex compatible with neuroinflammation, that was corrected with LGZ for both markers. Images were taken at a 10 × magnification (and 2.5 × electronic zoom for Iba1 images). Scale bar represents 50 µm. Statistical analyses were performed as described in Materials and Methods. *p < 0.05, **p < 0.01. Absence of asterisk means no statistically significant difference. Wild-type female mice in grey; Rett female mice in yellow; LGZ-treated Rett female mice in green

Previous reports had suggested an increased inflammatory signaling in Rett syndrome, detected in peripheral tissues in both Rett models and patient samples [26, 27]. Yet, the neuroinflammatory component of the disease had not been previously explored. To unveil its potential role in the pathogenesis of the disease, we performed a cytokine detection panel in symptomatic 7 months old Rett mice compared to age-paired controls. We observed a significant increase in the expression of several cytokines in Rett samples cortex compared to the wild-type (Fig. 5A, Additional file 1: Fig. S8A), although no differences were observed in cerebellum (Additional file 1: Fig. S8B). Since leriglitazone exerts an anti-inflammatory effect through NF-κB modulation, we studied its effect on the neuroinflammatory landscape in Rett syndrome, noting a correction in leriglitazone-treated animals (Fig. 5A). These results were ratified by the presence of microglia (through the expression of the marker Iba1) and mature astrocytes (through the marker GFAP) in Rett mice brains compared to wild-type, as we observed an increase in both GFAP and Iba1 in Rett mice cortex, which was significantly corrected through treatment with leriglitazone (Fig. 5B–E). Regarding the role of inflammation through neurodevelopment, we did not detect an inflammatory profile in pre-symptomatic mice (Additional file 1: Fig. S8C).

### PPARγ activation can result in the amelioration of the energetic deficits in other mitochondrial diseases

**Fig. 6:**
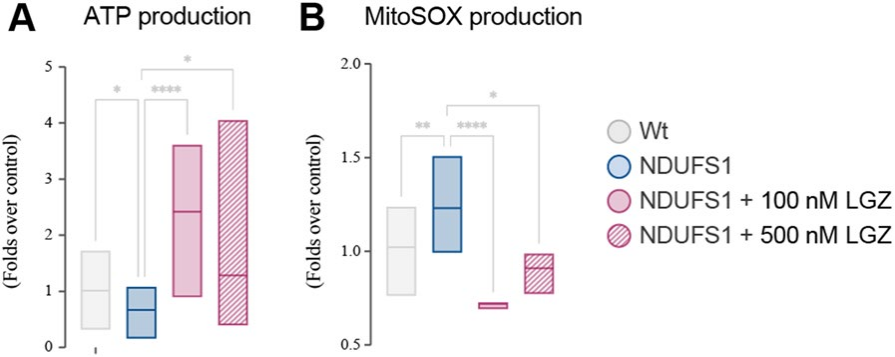
Effect of leriglitazone in fibroblasts from primary mitochondrial diseases. Given LGZ mechanism of action we investigated its potential effect on the treatment of mitochondrial diseases. For that, we selected fibroblasts from a patient bearing mutations in *NDUFS1*, which affect the bioenergetic function. **A** Non-glycolytic ATP concentration detected by luciferin-luciferase luminescence upon incubation with 2-deoxyglucose, and **B** O_2_^−^ generation—detected with MitoSOX probe by flow cytometry—were evaluated, confirming the effect of leriglitazone in mitochondrial patients. All experiments were done in at least two technical replicates and each experiment was done in triplicates. Statistical analyses were performed as described in Materials and Methods. *p < 0.05, **p < 0.01, ****p < 0.0001. Absence of asterisk means no statistically significant difference. Control fibroblasts in grey; NDUFS1-patient’s fibroblasts in blue; LGZ-treated NDUFS1-patient’s fibroblasts in rouge

After showing that leriglitazone can modulate mitochondrial dysfunction in Rett syndrome, we next asked whether it could be useful in the correction of mitochondrial deficits in the context of primary mitochondrial diseases. Mitochondrial diseases affect 1 in every 4300 people, and result from mutations in genes directly responsible for any of their functions, encoded either in mitochondrial or nuclear DNA. To test the effect of leriglitazone in primary mitochondrial disorders we have selected fibroblasts from a patient bearing a mutation in *NDUFS1*, which has been confirmed as the cause of the disease. We limited our study to the effect of leriglitazone in mitochondrial ATP production and ROS exposure, altered in the patient (Fig. 6A, B). Treatment with leriglitazone for 48 h effectively corrected the mitochondrial phenotype, increasing the mitochondria-derived ATP concentration in the patient fibroblasts, and significantly reducing the oxidative stress. These results suggest that leriglitazone could be efficient in the treatment of primary mitochondrial diseases in which bioenergetic functions are altered.

## Discussion

Rett syndrome is a devastating neurodevelopmental disease without specific treatment. While a disruption of excitatory/inhibitory neurotransmission stands out in its pathophysiology, mitochondrial dysfunction has been suggested to play a role in the disease development as well [6], and in the last years it is being proposed whether mitochondrial alterations can constitute a new therapeutic target for this disorder [7]. In our work, we have explored mitochondrial dysfunction focusing on mitochondrial shape and bioenergetic function. We have defined the specific alterations in patients’ fibroblasts and in Rett female mice brains, highlighting the brain-area specific differences and the changes that occur in the pre-symptomatic stages. Building on that, we have studied the effect of the novel drug leriglitazone on mitochondrial function enhancement, showing that it increases ATP production and reduces oxidative damage, ultimately improving the phenotype of symptomatic female mice. Our data endorse leriglitazone as a potential therapy not only for Rett syndrome, but also for other mitochondrial diseases.

Rett mitochondria form less interconnected and “simpler” less ramified networks with reduced fission: fusion ratio, along with several structural differences. These changes in cristae number and shape have been previously associated with reduced respiratory efficiency and cell viability, and reported in many diseases, including Leigh syndrome and Parkinson's disease [28, 29]. We have observed that Rett mitochondria produce half the ATP than controls do, also reflected in the respiration rates measured by Seahorse flux analytics, as the ATP-associated oxygen consumption is reduced with respect to controls. Not only Rett mitochondria produce less ATP, but they also generate more ROS (O_2_^·−^), resulting in an increased oxidative damage. These results build on previous studies reporting mitochondrial alterations in the context of Rett syndrome such as decreased respiration and ATP production [30, 31], enhanced oxidative stress [32] or mitochondrial elongation [19]. They also confirm fibroblasts as a valid, and understudied, model for the research on mitochondrial alterations in this disease, enabling the study patient-specific differences. Further works shall explore the potential correlation between patients and fibroblasts response to a drug, advancing towards personalized medicine in Rett syndrome.

The bioenergetic alterations are maintained in Rett mice brains. This agrees with studies that reported a decrease in brain ATP synthesis [33–35], but while knowledge was mostly limited to whole brain analysis, we have studied bioenergetic function both between different brain areas and at two different time-points through the phenotype progression. Our results in symptomatic 7 m.o. Rett mice evidence the diversity on mitochondrial performance through the brain, and point to the cerebellum as a major player in the pathophysiology of Rett syndrome with respect to bioenergetic dysfunction. Previous results reported an increase in the rate of H_2_O_2_ generation due to dysfunctional complex II in Rett whole brain [36], which would be expected to result in an increase in lipid peroxidation. Opposite to that, we have observed a decrease in lipid peroxidation in both hippocampus and cerebellum of Rett mice and an increase in cortex. This discrepancy might be sustained on two aspects: on one hand, cortex is the most voluminous area in the brain, and therefore the results in that area might overshadow the differences in other brain areas. On the other hand, previous reports suggest an alteration in lipids metabolism, profiled both in Rett mice [24, 37, 38] and patient’s cerebrospinal fluid [25], probably affecting the overall lipid peroxidation detection. Clarifying this, the increase in 8-OHdG in cerebellum supports oxidative damage is also involved in brain mitochondrial dysfunction, in agreement with previous reports. Cerebellar bioenergetic alterations are already detectable in pre-symptomatic phases (3 m.o. mice), underlining its importance in disease progression. Alterations in cerebellum function had been suggested in Rett patients through MRI [39], and recent experiments show that deleting Mecp2 from the cerebellum is responsible for the delay in motor learning in mice [40]. In fact, a parallelism could be established between cerebellar dysfunctions and early-presenting features of the disease such as the movement disorders [41] or stereotypes [42]. Despite all this, cerebellum remains understudied in Rett syndrome compared to other brain areas as cortex or hippocampus [40]. This gap in the knowledge is bigger if we focus in bioenergetic performance, that can be playing an even more important role in cerebellum than in other brain areas. Taking cerebellum into consideration as an important player in the pathophysiology of Rett syndrome is especially relevant on the design of new therapies, such as transcranial electrical stimulation (tES) [43, 44] or genetic therapy, where reach of the transgene can be a limitation [45].

This systematic description and delineation of the involvement of mitochondria in Rett syndrome’s pathophysiology sets the base for its study as a potential therapeutic target for the treatment of the disease. While this had already been attempted with other drugs, the effects observed were mild and have not been clinically translatable [10, 11, 46]. Building on that, we have studied the effect of the novel drug leriglitazone on the correction of Rett dysfunctions. Leriglitazone (LGZ) is an oral bioavailable selective PPARγ agonist that has an improved profile compared with other drugs acting on the same target for treating neurodegenerative diseases. It shows superior brain penetration and a favorable safety profile, allowing interaction with PPARγ in the CNS above the level that can be safely achieved with pioglitazone and other glitazones [13]. It promotes the transcription of key genes that counteract oxidative stress, stimulate mitochondrial biogenesis and repress Nf-kB activation [22]. It has been validated in preclinical models of other neurodegenerative diseases, showing clinical benefits for X-ALD patients and clinical proof of concept in FA patients [13, 14, 22, 23, 47].

Treatment with leriglitazone restores mitochondrial homeostasis in Rett models. Our studies show that LGZ increases mitochondrial ATP production and reduces oxidative stress, which can be defined as an improved bioenergetic status. These effects were recorded both in fibroblasts and in animal models, in which leriglitazone recovered the decrease in ATP concentration and normalized lipid peroxidation profiles and oxidative damage markers both in hippocampus and cerebellum.

On top of mitochondrial homeostasis correction, leriglitazone also reduced inflammation in Rett mice. Inflammatory involvement in the disease had been previously pointed out in patients’ plasma and fibroblasts [26, 48], and this is the first report on a neuroinflammatory activity in Rett brain, evidenced by the cytokine panel and the increase in microglia in cortex. Surprisingly, while bioenergetic alterations were more prominent in cerebellum and already detectable at a pre-symptomatic stage, inflammatory alterations were only noticeable in symptomatic brain cortex. Although mitochondrial dysfunction has recently been proposed as an inflammatory trigger in several scenarios [49, 50], our results suggest that mitochondrial dysfunction and neuroinflammation are not necessarily interrelated in Rett syndrome and nor is their correction with leriglitazone. Neuroinflammatory component of the disease sets a new therapeutic target for Rett syndrome. Further studies shall investigate its contribution to the disease progression.

Importantly, sustained treatment with leriglitazone results in an amelioration of the phenotype of female Rett mice, both in the general observed health and in the exploratory activity. Similar phenotypic modulation through mitochondrial targeting had been previously achieved through serotonin receptor stimulation [10, 46], but not accomplished through other approaches as metformin [11]. We believe the success in leriglitazone effect in Rett syndrome relies on both the treatment time (from weaning until sacrifice) and on the mechanism of action of leriglitazone, increasing mitochondrial performance and biogenesis, exerting an anti-inflammatory effect and promoting neuronal growth and myelination [13]. Working with female mice greatly increases the translational value of these results.

Given the effect of leriglitazone on mitochondrial function enhancement, and its prompt availability in other neuropediatric diseases, we wondered whether it will be effective on primary mitochondrial diseases. To such end, we treated with leriglitazone fibroblasts from mitochondrial patients bearing a mutation in *NDUFS1*, which showed alterations affecting directly the electron transport chain. Treatment with leriglitazone resulted in an increase in ATP concentration and decreased oxidative stress, outstanding as a potential treatment for mitochondrial diseases.

Taken together, our work provides evidence of mitochondrial dysfunction in Rett syndrome, sustained from patient fibroblasts to Rett female mice, complementing previous reports on total brain metabolic defect and highlighting the importance of the cerebellum. Noteworthy, cerebellar mitochondrial dysfunction is already evident at the pre-symptomatic stage, which underlines the importance of timing in the study and treatment of Rett syndrome. Our results confirm that Rett syndrome is indeed a neurodevelopmental disorder with a key metabolic component, which can be purposed as a therapeutic target, urging for a prompt mitochondria-targeted intervention upon disease diagnosis.

The bioenergetic and inflammatory alterations are corrected in the studied models through treatment with the PPARγ agonist leriglitazone, resulting in a phenotypic improvement of Rett female mice. Given these results and the knowledge on leriglitazone safety on both healthy subjects and pediatric patients [13], this work constitutes the preclinical evidence for a clinical trial with leriglitazone in Rett syndrome and other diseases with mitochondrial involvement.

## Conclusions

- Both mitochondrial shape and bioenergetic functions are altered in Rett syndrome fibroblasts, which display simpler and less interconnected networks, with defective ATP production and increased oxidative stress.
- The bioenergetic dysfunction is also detectable in Rett syndrome female mice models, displaying differences among brain areas and already evident at pre-symptomatic stages.
- Cerebellum, an understudied area in the pathophysiology of Rett syndrome, outstands regarding the bioenergetic dysfunction.
- Leriglitazone corrects bioenergetic alterations in all the studied Rett models, together with the amelioration of the phenotypic outcome in symptomatic Rett female mice.
- Rett syndrome pathophysiology includes a neuroinflammatory component, which is prevented with leriglitazone.
- This work provides preclinical evidence foundational for a clinical trial with leriglitazone for the treatment of Rett syndrome.

### Supplementary Information

**Additional file 1: Table S1**. MeCP2 mutations correspondent to each studied patients’ fibroblasts. **Figure S1**. Individualities between Rett patients’ fibroblasts regarding mitochondrial network and bioenergetics. **Figure S2**. ATP production in brain areas through development.** Figure S3**. Leriglitazone (LGZ) upregulates genes under the PPARγ pathway and exerts an effect on mitochondrial biogenesis, without changes in antioxidant markers expression. **Figure S4**. Leriglitazone (LGZ) does not exert an effect on mitochondrial dynamics in Rett fibroblasts. **Figure S5**. Blocking PPARγ pathway with GW9662 results in a decrease in ATP production, without changes in superoxide anion generation. **Figure S6. **LGZ plasma detection in symptomatic Rett female mice. **Figure S7**. Oxidative stress in cerebellum of symptomatic Rett female mice. **Figure S8**. Neuroinflammatory component is not detected in cerebellum of symptomatic mice nor presymptomatic cerebral cortex.

**Additional file 2: Video S1.** Time-lapse video of mitochondrial network dynamics of control fibroblasts (2 minutes, scale bar 10 μm).

**Additional file 3: Video S2**. Time-lapse video of mitochondrial network dynamics of Rett fibroblasts (2 minutes, scale bar 10 μm).

**Additional file 4: Video S3**. Time-lapse video of mitochondrial network dynamics of LGZ-treated Rett fibroblasts (2 minutes, scale bar 10 μm).

## Data Availability

The datasets used and/or analysed during the current study are available from the corresponding author on reasonable request.

## Abbreviations

8-OHdG: 8-Hydroxy-2ʹ-deoxyguanosine
Drp1: Dynamin-related protein 1
FA: Friedreich’s ataxia
Fis1: Mitochondrial fission 1 protein
GPX: Glutathione peroxidase 1
MECP2: Methyl-CpG binding protein 2
Mfn2: Mitofusin 2
MnSOD: Manganese superoxide dismutase
OPA1: OPA1 mitochondrial dynamin like GTPase
PGC1α: Peroxisome proliferator activated receptor gamma coactivator 1
PPARγ: Peroxisome proliferator-activated receptor-gamma
TOMM20: Translocase of the outer mitochondrial membrane complex subunit 20.
XALD: X-linked adrenoleukodystrophy
