# Supplementary material for: Mitochondrial modulation with leriglitazone as a potential treatment for Rett syndrome
Source: J Transl Med. 2023 Oct 26;21:756. doi: 10.1186/s12967-023-04622-5 (PMC10601217; doi:10.1186/s12967-023-04622-5)
Supplement: Supplementary file 1 — Additional file 1: Table S1. MeCP2 mutations correspondent to each studied patients’ fibroblasts. Figure S1. Individualities between Rett patients’ fibroblasts regarding mitochondrial network and bioenergetics. Figure S2. ATP production in brain areas through development. Figure S3. Leriglitazone (LGZ) upregulates genes under the PPARγ pathway and exerts an effect on mitochondrial biogenesis, without changes in antioxidant markers expression. Figure S4. Leriglitazone (LGZ) does not exert an effect on mitochondrial dynamics in Rett fibroblasts. Figure S5. Blocking PPARγ pathway with GW9662 results in a decrease in ATP production, without changes in superoxide anion generation. Figure S6. LGZ plasma detection in symptomatic Rett female mice. Figure S7. Oxidative stress in cerebellum of symptomatic Rett female mice. Figure S8. Neuroinflammatory component is not detected in cerebellum of symptomatic mice nor presymptomatic cerebral cortex. [file 12967_2023_4622_MOESM1_ESM.pdf]

Table S1. MeCP2 mutations correspondent to each studied patients' fibroblasts. It is specified the systematic name of each mutation, the amino acid change, the percentage of this mutation corresponding to the RettBASE, and the protein affected domain. NLS: nuclear localization sequence; TRD: transcription repression domain; MDB: methyl-DNA binding.

Patient #	Systematic name	Amino acid change	Relative frequency[1]	Domain
**1**	c.502C>T	p.Arg168*	7.57	Inter-domain region
**2**	c.916C>T	p.Arg306Cys	5.14	TRD
**3**	c.502C>T	p.Arg168*	7.57	Inter-domain region
**4**	c.423C>G	p.Tyr141*	0.33	MBD
**5**	c.423C>G	p.Tyr141*	0.33	MBD
**6**	c.880C>T	p.Arg294*	4.97	TRD
**7**	c.808C>T	p.Arg270*	5.74	NLS
**8**	c.763C>T	p.Arg255*	6.64	TRD

Reference:

1. Christodoulou J, Grimm A, Maher T, Bennetts B. RettBASE: The IRSA MECP2 variation database-a new mutation database in evolution. *Hum Mutat*. 2003 May;21(5):466-72. doi: 10.1002/humu.10194.

Relative frequency defined as the percentage of a specific mutation relative to all mutations described in *MECP2*.

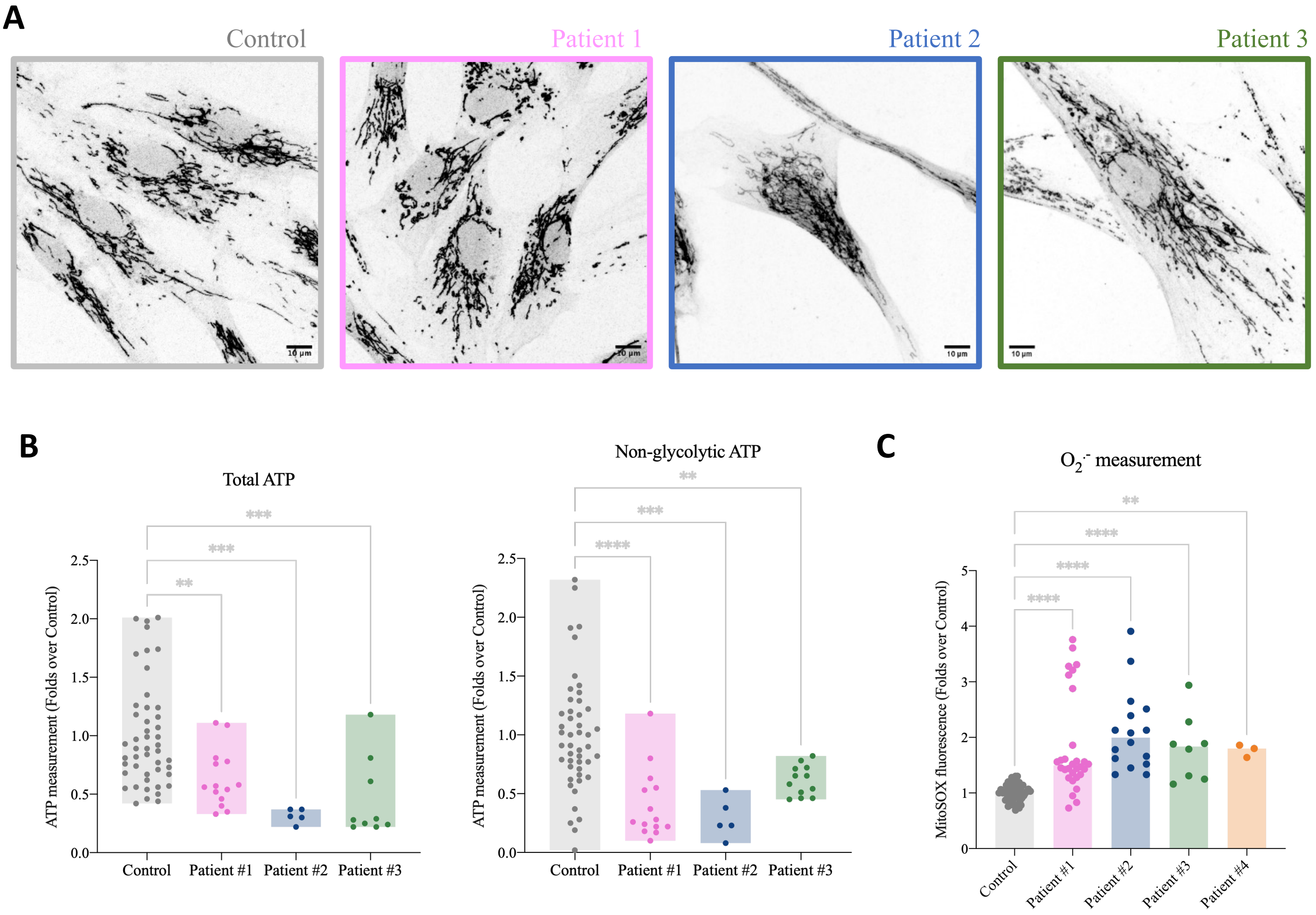

Figure S1. Individualities between Rett patients' fibroblasts regarding mitochondrial network and bioenergetics. (**A**) Mitochondrial network differs between Rett patients' fibroblasts (TOMM20 in black; scale bar 10 µm). (**B**) Total and non-glycolytic (mitochondrial) ATP production in three different patients' fibroblasts, detected by luciferin-luciferase luminiscence, and (**C**) superoxide anion measurement, detected by flow cytometry with MitoSOX Red probe, in four patients' fibroblasts with different mutations. **= *p*-value<0.01, ***= *p*-value<0.001, ****= *p*-value<0.0001.

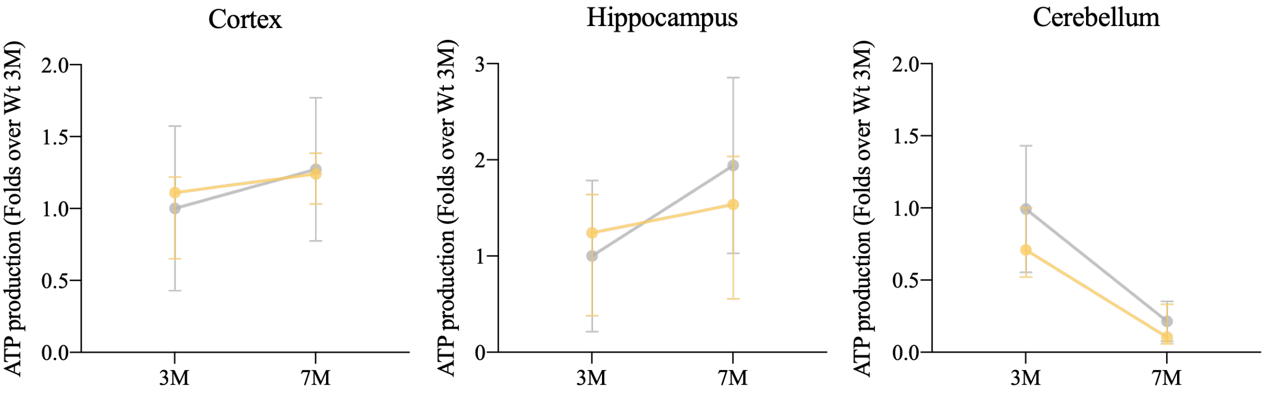

Figure S2. ATP production in brain areas through development. ATP concentrations have been compared between both timepoints in the three different areas, showing the differences between Rett and control samples.

Grey lines and dots show control values and yellow show Rett.

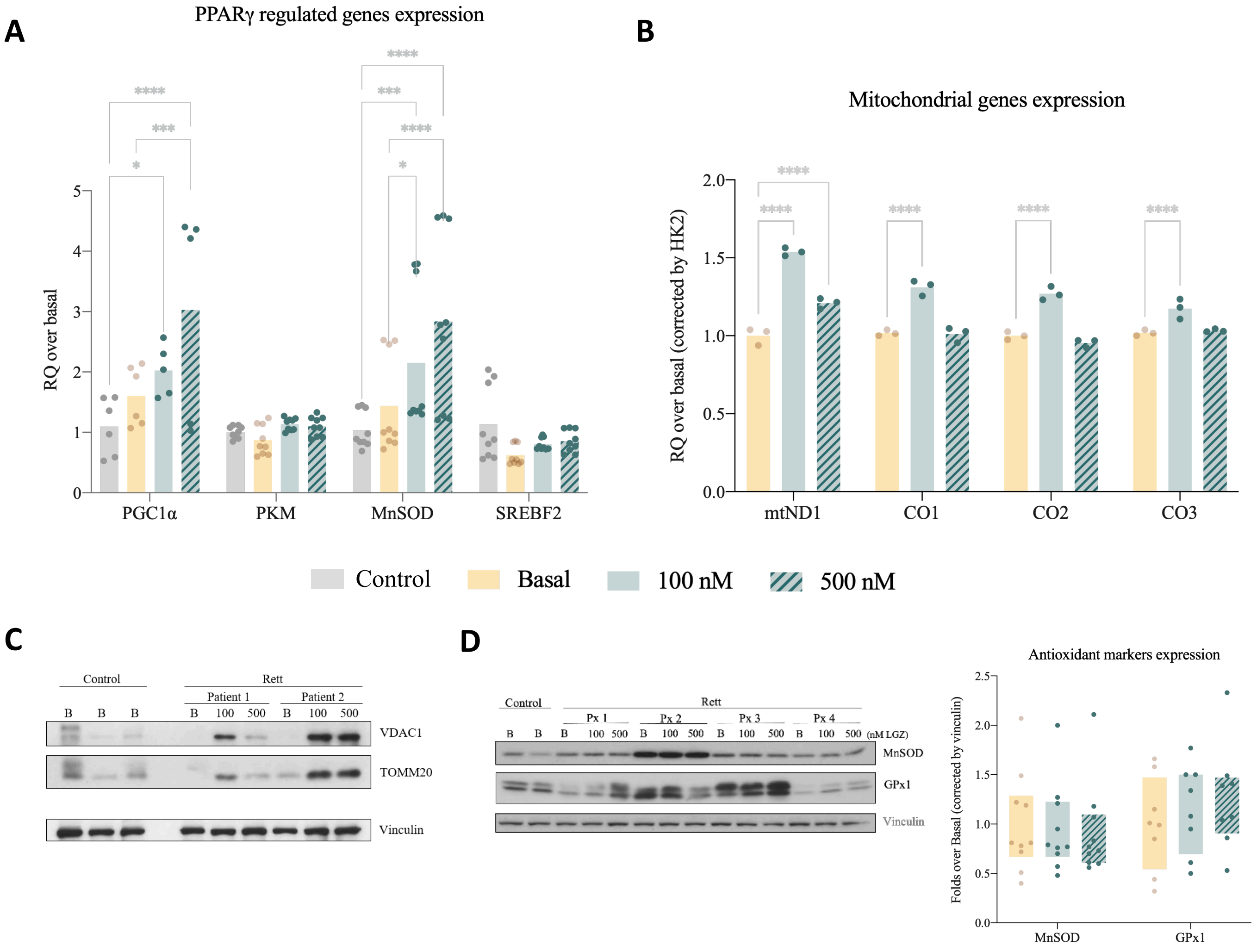

Figure S3. Leriglitazone (LGZ) upregulates genes under the PPARγ pathway and exerts an effect on mitochondrial biogenesis, without changes in antioxidant markers expression. (**A**) Appraisal of the genes regulated by the PPARγ activation pathway: *PGC1α*, *PKM*, *MnSOD*, and *SREBF2*, measured by qPCR. (**B**) PPARγ activation is expected to result in an increase in mitochondrial biogenesis, which was estimated by quantification of *mtND1, CO1, CO2* and *CO3* gene expression over *HK2*, measured by qPCR. (**C**) Mitochondrial proteins VDAC1 and TOMM20 expression is also increased after LGZ treatment in Rett fibroblasts, detected by Western blot. (**D**) Analysis and densitometry quantification of antioxidant markers MnSOD and GPX expression detected by Western blot and corrected by vinculin. *= *p*-value<0.05, ***= *p*-value<0.001, ****= *p*-value<0.0001.

Grey bars and dots show control values, yellow show Rett and green show leriglitazone-treated.

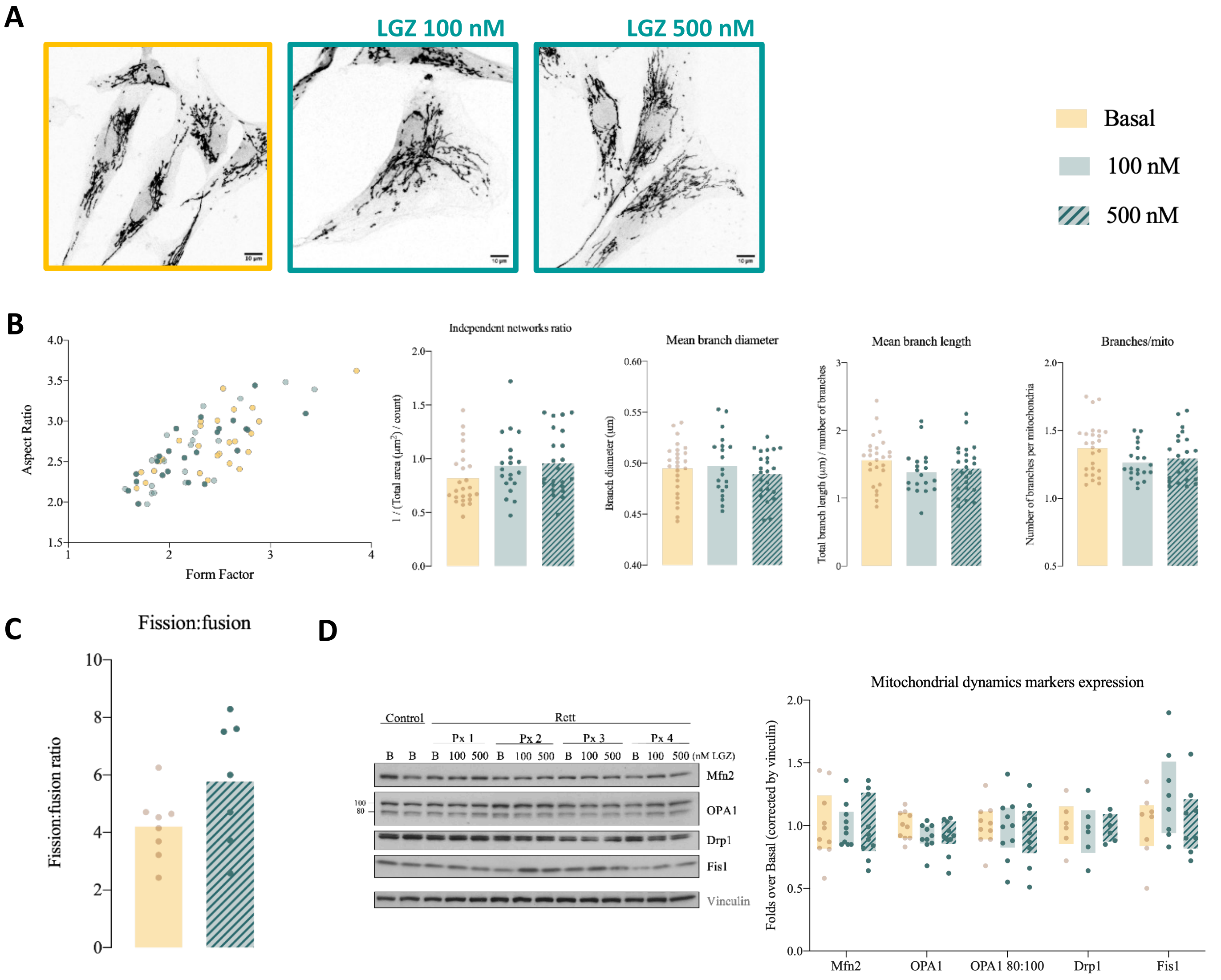

Figure S4. Leriglitazone (LGZ) does not exert an effect on mitochondrial dynamics in Rett fibroblasts. (**A**) Mitochondrial network and its morphological and branching analysis (**B**) are unaltered in Rett fibroblasts after LGZ (TOMM20 in black; scale bar 10 µm), analyzed with the Fiji plug-in Mitochondria Analyzer. (**C**) Fission: fusion ratio is not corrected after LGZ treatment, detected with MitoTracker Green and calculated from 2-minute time-lapse images using the MATLAB add-in Mitometer. (**D**) Analysis and densitometry quantification of mitochondrial dynamics markers detected by Western blot, and corrected by vinculin..

Yellow bars and dots show Rett and green show leriglitazone-treated.

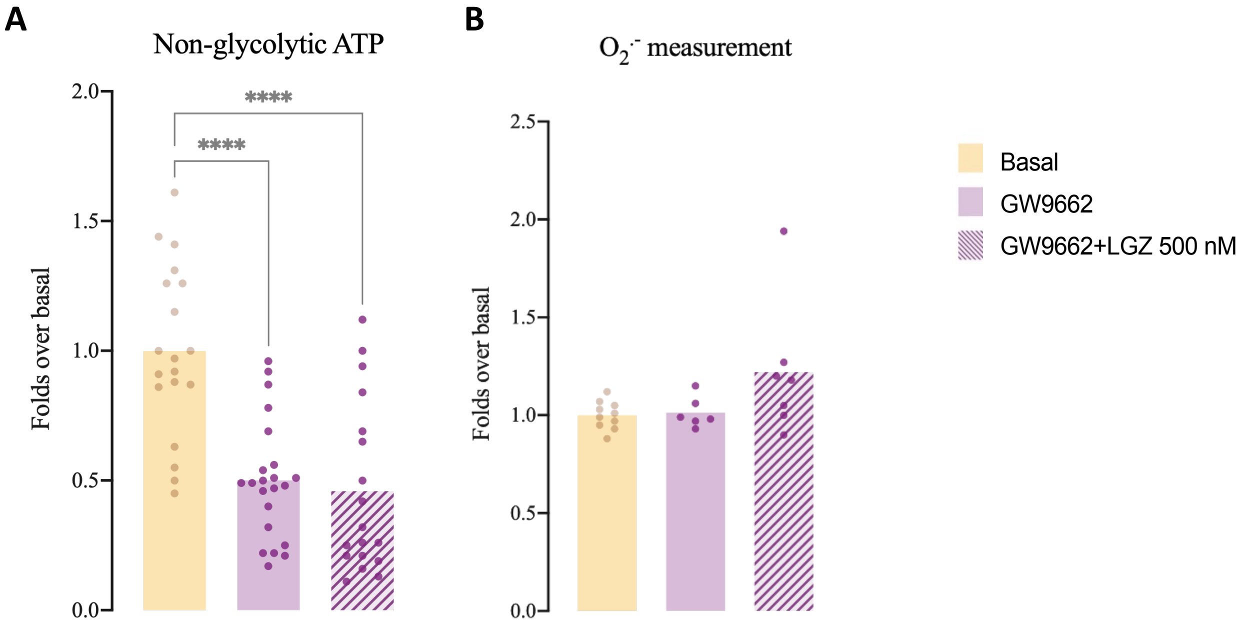

Figure S5. Blocking PPARγ pathway with GW9662 results in a decrease in ATP production, without changes in superoxide anion generation. (**A**) Non-glycolytic (mitochondrial) ATP production, detected by luciferin-luciferase luminiscence, is decreased in Rett fibroblasts treated with the PPARγ antagonist GW9662 at 1 µM for 72h, and not recovered when treated with a combination of GW9662 1 µM + LGZ 500 nM. (**B**) Superoxide anion generation, detected by flow cytometry with MitoSOX Red probe, is not changed when cells are treated with GW9662 nor together with the combination of combination of GW9662 1 µM + LGZ 500 nM. ****= *p*-value<0.0001.

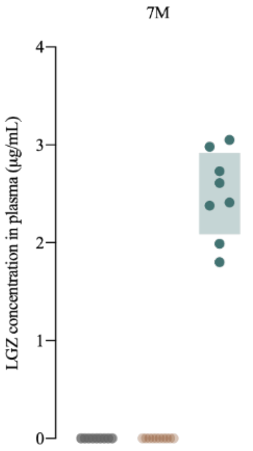

Figure S6. LGZ plasma detection in symptomatic Rett female mice. LGZ plasma concentrations detected by HPLC only in treated mice. Grey dots show wild-type female mice; yellow Rett mice; and green leriglitazone-treated Rett mice.

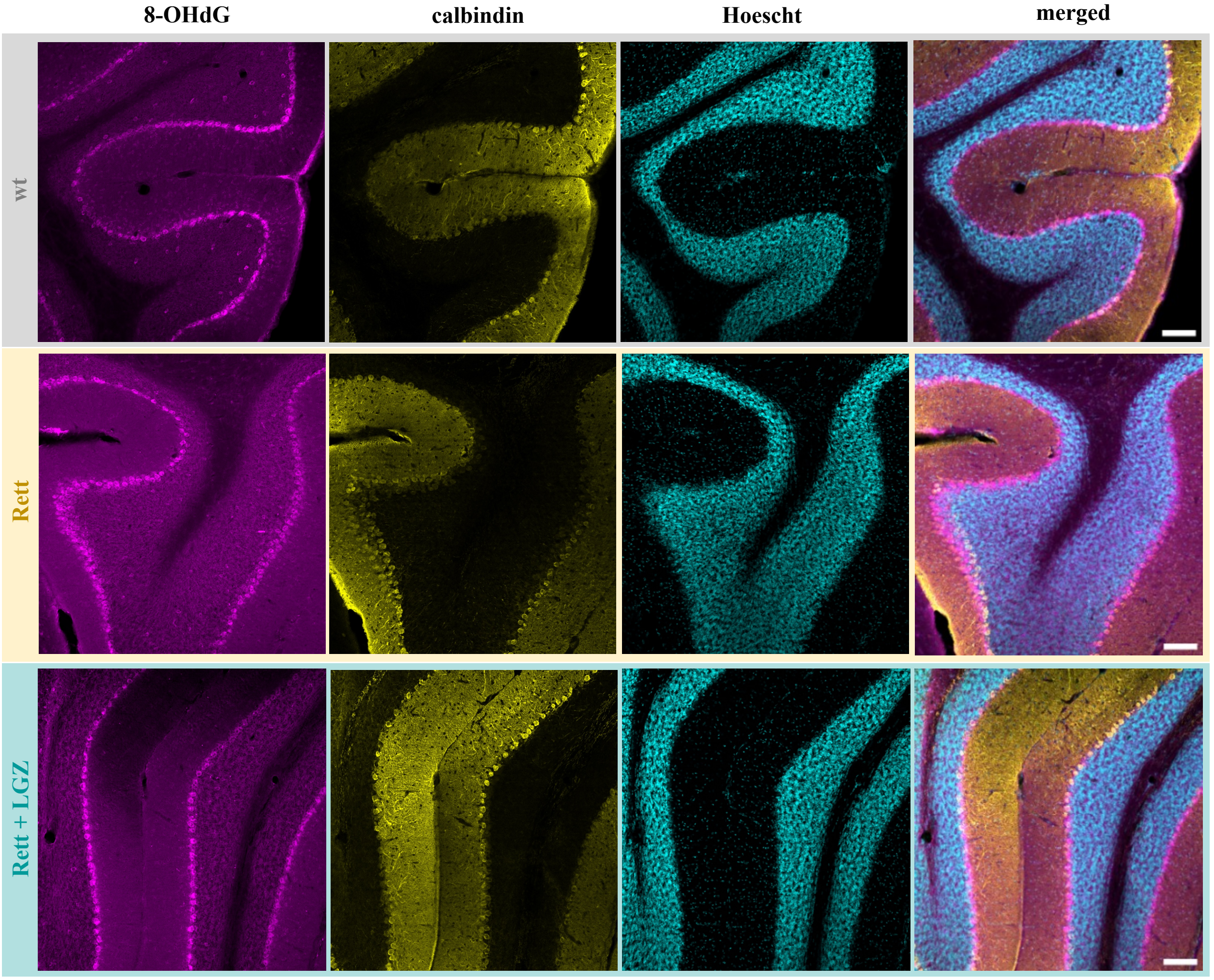

Figure S7. Oxidative stress in cerebellum of symptomatic Rett female mice. Representative images showing the RNA oxidative marker 8-OHdG in magenta, Purkinje cells marker calbindin in yellow and Hoescht for nuclei in cyan; scale bar 100 µm.

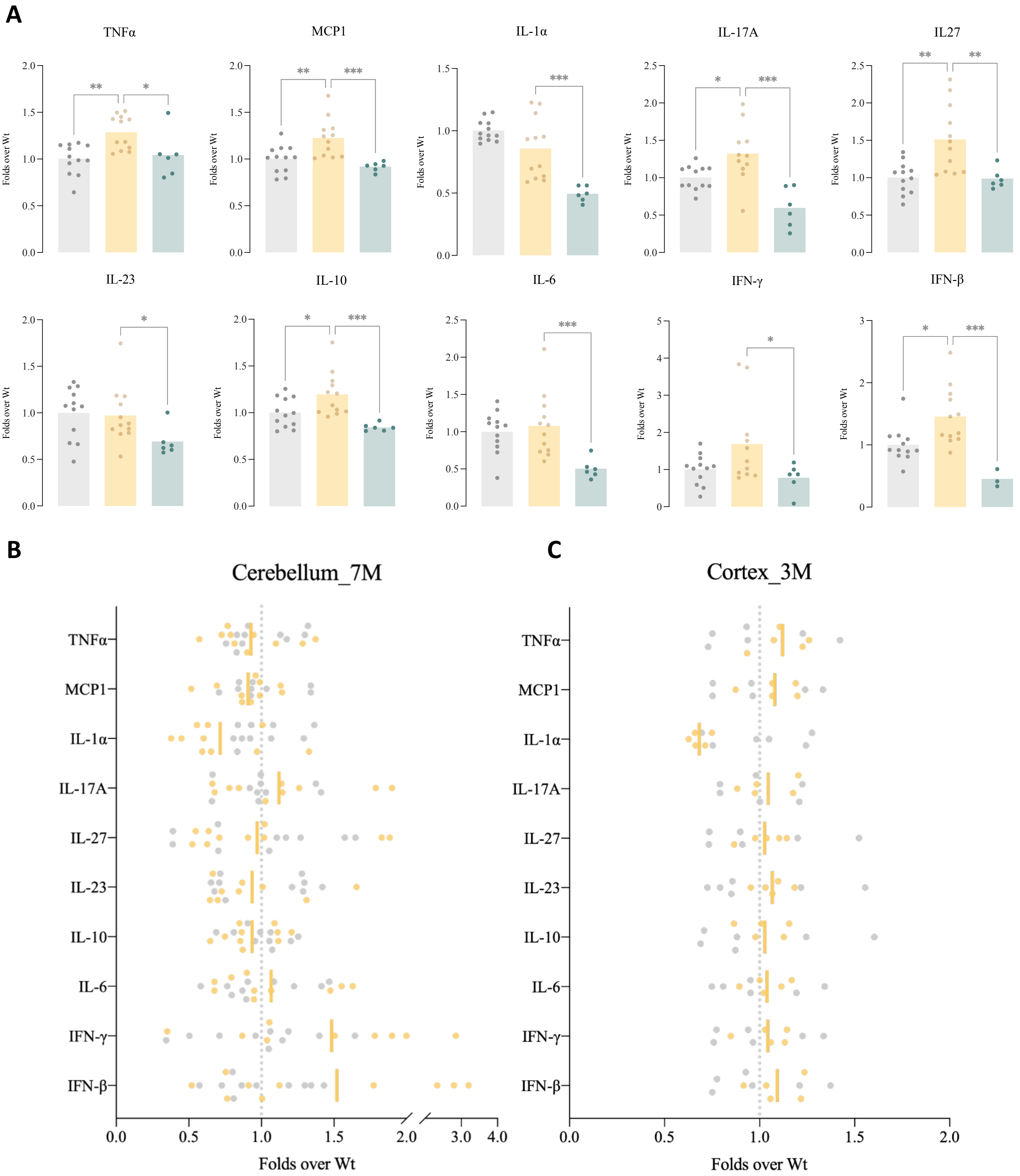

Figure S8. Neuroinflammatory component is not detected in cerebellum of symptomatic mice nor presymptomatic cerebral cortex. **(A)** Analysis and representation of each cytokine individually in symptomatic mice cortex. Cytokine detection by flow cytometry in (**B**) cerebellum of 7 m.o. mice and in (**C**) cortex of 3 m.o. mice. Nested t-test analysis of these cytokines shows that there are no differences between the two groups (Wt and Rett mice).

Grey bars and dots show wild-type mice values, yellow show Rett and green leriglitazone-treated.

(*P* value = 0.7111 for cerebellum_7m ; *P* value = 0.5844 for cortex_3m).
